# Transcriptional profiling of sweetpotato (*Ipomoea batatas*) roots indicates down-regulation of lignin biosynthesis and up-regulation of starch biosynthesis at an early stage of storage root formation

**DOI:** 10.1186/1471-2164-14-460

**Published:** 2013-07-09

**Authors:** Nurit Firon, Don LaBonte, Arthur Villordon, Yanir Kfir, Julio Solis, Evgenia Lapis, Temima Schnitzer Perlman, Adi Doron-Faigenboim, Amots Hetzroni, Leviah Althan, Lahan Adani Nadir

**Affiliations:** 1 Institute of Plant Sciences, The Volcani Center, Agricultural Research Organization, Bet Dagan 50250, Israel; 2 LSU AgCenter School of Plant, Environmental, and Soil Sciences, 137 J.C. Miller Hall, Baton Rouge, LA 70803-2120, USA; 3 LSU AgCenter Sweet Potato Research Station, Chase, LA 71324, USA; 4 Medical Goldyne Savad Institute of Gene Therapy, Hadassah Medical Center, Jerusalem 91120, Israel; 5 Agricultural Engineering, Sensing, Information and Mechanization Engineering, The Volcani Center, Agricultural Research Organization, Bet Dagan 50250, Israel

**Keywords:** Fibrous root, *Ipomoea batatas*, Lignin biosynthesis, Starch biosynthesis, Storage-root initiation, Transcription profiling

## Abstract

**Background:**

The number of fibrous roots that develop into storage roots determines sweetpotato yield. The aim of the present study was to identify the molecular mechanisms involved in the initiation of storage root formation, by performing a detailed transcriptomic analysis of initiating storage roots using next-generation sequencing platforms. A two-step approach was undertaken: (1) generating a database for the sweetpotato root transcriptome using 454-Roche sequencing of a cDNA library created from pooled samples of two root types: fibrous and initiating storage roots; (2) comparing the expression profiles of initiating storage roots and fibrous roots, using the Illumina Genome Analyzer to sequence cDNA libraries of the two root types and map the data onto the root transcriptome database.

**Results:**

Use of the 454-Roche platform generated a total of 524,607 reads, 85.6% of which were clustered into 55,296 contigs that matched 40,278 known genes. The reads, generated by the Illumina Genome Analyzer, were found to map to 31,284 contigs out of the 55,296 contigs serving as the database. A total of 8,353 contigs were found to exhibit differential expression between the two root types (at least 2.5-fold change). The Illumina-based differential expression results were validated for nine putative genes using quantitative real-time PCR. The differential expression profiles indicated down-regulation of classical root functions, such as transport, as well as down-regulation of lignin biosynthesis in initiating storage roots, and up-regulation of carbohydrate metabolism and starch biosynthesis. In addition, data indicated delicate control of regulators of meristematic tissue identity and maintenance, associated with the initiation of storage root formation.

**Conclusions:**

This study adds a valuable resource of sweetpotato root transcript sequences to available data, facilitating the identification of genes of interest. This resource enabled us to identify genes that are involved in the earliest stage of storage root formation, highlighting the reduction in carbon flow toward phenylpropanoid biosynthesis and its delivery into carbohydrate metabolism and starch biosynthesis, as major events involved in storage root initiation. The novel transcripts related to storage root initiation identified in this study provide a starting point for further investigation into the molecular mechanisms underlying this process.

## Background

Sweetpotato [*Ipomoea batatas* (L.) Lam.] is the seventh most important food crop in the world [1]. It has a worldwide production of approximately 107 million metric tons and is the third most important root/tuber crop after potato (*Solanum tuberosum* L.) and cassava (*Manihot esculenta* Crantz). World production is centered in Southeast Asia, with China being the largest producer, while Sub-Saharan Africa ranks second [2]. Sweetpotato is used as a source of starch, ethanol, and animal fodder in most of Asia while it is considered a subsistence crop in Africa. The USA, Japan, Australia, New Zealand, South Africa and Israel are among the few producing countries that grow sweetpotato as a vegetable to market in developed economies. Global demand for sweetpotato is on the rise due to its high nutritional value. The *Nutrition Action Health Letter* from the Center for Science in the Public Interest, USA [3], rates sweetpotatoes as the highest ranking vegetable for human nutrition.

The most economically important physiological process in sweetpotato production is storage root (SR) development. Sweetpotatoes are commercially propagated through vegetative cuttings. These cuttings produce adventitious roots that give rise to the SRs [4-7]. Adventitious roots originate from primordia located on the nodes, as well as from the cut ends, i.e., “wound” roots [4,6]. Initially, white fibrous roots (FRs) develop and some of these subsequently develop into SRs. Depending on the number of FRs induced to form SRs, sweetpotato plants will yield either a high (4 to 8 per plant) or low number of SRs that may even be reduced to one very large SR per plant [7]. Togari [4] described the sequence of anatomical events leading to SR initiation in varieties *Okinawan* and *Beinakzi* and reported that the regular vascular cambium layer first appears 20 days after transplanting (DAT), followed by the initial development of secondary anomalous cambium (AC) features at 25 DAT. Togari [4] also documented the incidence of stele lignification and proposed that lignification prevents SR initiation. Wilson and Lowe [5] also suggested that only the appearance of AC can prevent stele lignification.

Recently, we demonstrated in both *Georgia Jet* and *Beauregard* sweetpotato varieties (the leading Israeli variety and an important USA variety, respectively), that the period spanning 5 to 35 DAT is critical in determining whether adventitious roots become lignified or initiate as SRs, and that the appearance of AC marks the initial phase of SR formation [7]. The molecular mechanisms underlying the induction of adventitious roots to become SRs are, however, poorly understood. Expression studies have been used in an effort to elucidate factors involved in SR formation. You et al. [8] constructed a cDNA library of early-stage SRs, and identified 22 genes differentially expressed between FRs (non-storage and storage stage) and SRs. Among them were a no apical meristem (NAM)-like and a MADS-box (MCM1, AGAMOUS, DEFICIENS and SRF) protein gene, both of which were down-regulated in SRs. McGregor [9] found several NAC family transcriptional regulator proteins that were down regulated in storage roots, similar to the NAM-like protein described by You et al. [8]. McGregor [9] also identified up-regulated expression of two NAM-like genes, as well as sporamin genes and genes involved in starch biosynthesis, in storage roots that developed six weeks after planting compared to fibrous roots. Several additional MADS-box genes (*IbMADS1*, *IbMADS3*, *IbMADS4*, *IbMADS10*, *IbMADS79*, *IbAGL17*, *IbAGL20* and *SRD1*) expressed in root tissues have been isolated from sweetpotato, and their possible roles in root development have been deduced [10-15]. Tanaka et al. [16] identified 10 genes with differential expression among FRs, thick roots, and SRs. One of the genes, *SRF6*, encoded a receptor-like kinase with high expression around the primary cambium and xylem meristem. In addition, Tanaka et al. [17] suggested three sweetpotato class 1 knotted1-like homeobox (*KNOX1*) genes as possible regulators of cytokinin levels in SRs.

De-novo assembly of transcript sequences produced by next-generation sequencing (NGS) technologies offers a rapid approach to obtaining expressed gene sequences for non-model organisms. Indeed, most recently, NGS was used by several research groups to obtain leaf, stem and root transcriptome data for different sweetpotato cultivars [18-20]. Tao et al. [20] used Illumina NGS, employing a combination of different tissues at different developmental stages, to generate 51,736 annotated transcripts, and identified differentially expressed transcripts in different tissues, including roots. Xie et al. [21] analyzed the transcriptome of a purple sweetpotato, obtaining a total of 58,800 unigenes, and suggested UDP-glucose-flavonoid 3-O-glucosyltransferase as one of the key enzymes in anthocyanin biosynthesis and that anthocyanin-3-glucoside might be one of the major components for anthocyanin pigments in the purple sweetpotato.

The presently described study focused on the identification of the molecular mechanisms involved in the initiation of SR formation in the leading sweetpotato variety in Israel, *Georgia Jet*, by performing a detailed transcriptomic analysis of initiating SRs (ISRs) using NGS platforms. A two-step approach was undertaken: (1) generating a database for the sweetpotato root transcriptome using 454-Roche sequencing of a cDNA library created from pooled samples of two root types: FRs and ISRs; (2) comparing the expression profiles of ISRs and FRs, using the Illumina Genome Analyzer to sequence non-normalized cDNA libraries of the two root types and mapping the data onto the root transcriptome database. Use of the 454-Roche platform generated a total of 524,607 reads, 85.6% of which were clustered into 55,296 contigs that matched 40,278 known genes. The differential expression profiles between the two root types obtained by the Illumina platform indicated down-regulation of classical root functions, such as transport and response to the environment, and of lignin biosynthesis in ISRs, along with up-regulation of carbohydrate metabolism and starch biosynthesis. In addition, the data suggest delicate control of stem cell maintenance and differentiation in sweetpotato vascular development associated with the initiation of SR formation.

## Results and discussion

### Insight into the transcriptome of sweetpotato roots

#### ***Defining the transcriptome using 454 sequencing and de-novo assembly***

**Figure 1:**
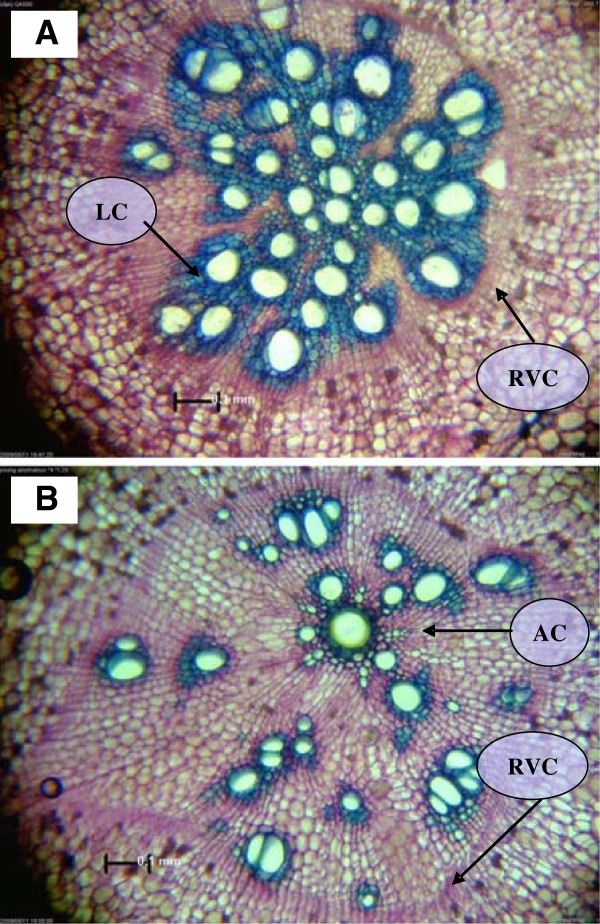
**Microscopic view of adventitious root cross sections 26 days after transplanting. A**. Fibrous root (FR) cross section. **B**. Initiated storage root (ISR) cross section. Tissue sections were taken at 3 cm from the proximal root part and stained with toluidine blue. LC – lignified cells; AC – anomalous cambium; RVC – regular vascular cambium. Scale bars = 0.1 mm.

To obtain insight into the molecular mechanisms involved in the initiation of SR formation in sweetpotato, and to identify candidate genes involved in this process, a two-stage approach was adopted. First, a database of the sweetpotato (var. *Georgia Jet*) root transcriptome was generated, using 454-Roche sequencing of a cDNA library created from pooled RNA samples of two root types: ISRs and non-initiating FRs. Roots were divided into either ISRs or FRs following microscopic analysis, as shown in Figure 1. Second, the expression profiles of ISRs and FRs were compared, using the Illumina Genome Analyzer, to sequence non-normalized cDNA libraries of the two root types and the data were mapped onto the root transcriptome database.

**Figure 2:**
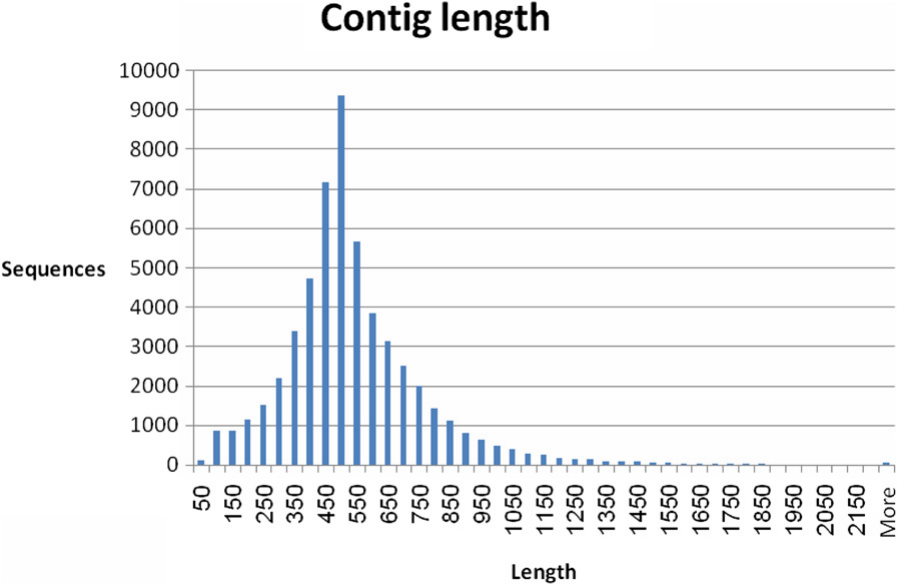
Sequence length distributions of contig sequences in the sweetpotato root transcriptome.

A total of 524,607 high-quality reads were generated with an average read length of 310 bp. The total number of bases (without keys, tags or bad-quality bases) was 1.63E + 08. The MIRA assembly clustered 85.6% (449,282 sequences) of the 454 sequence reads into 55,296 contigs. The sequence length distribution is illustrated in Figure 2. The average length of the contigs was 519 ± 245 bases. The remaining 75,325 (orphan) sequences were retained as singletons. The clustered contig data are available via a web page connected to The Volcani Center, Agricultural Research Organization web page at http://batata.agri.gov.il/.

#### ***Functional annotation by sequence comparison with public databases***

**Figure 3:**
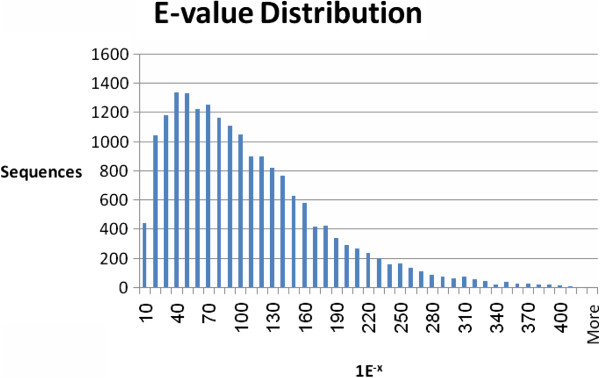
E-value distribution of contig sequences’ BLAST results in the sweetpotato root transcriptome.

**Figure 4:**
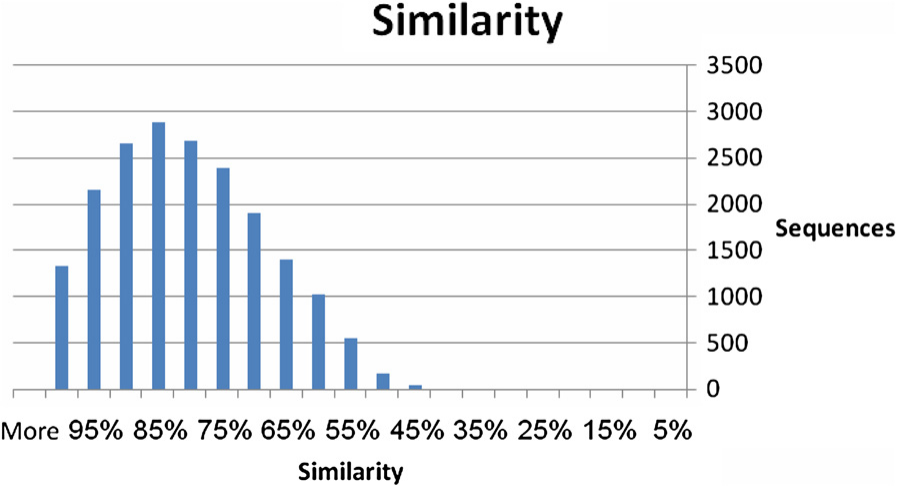
Similarity distributions of the contigs to their BLAST results.

The transcriptomic data were used to query public genomic databases [i.e. NCBI non-redundant (Nr)] using BLASTX. Of the 55,296 contigs, 40,278 (73%) matched known genes at a cut-off E value ≤ 1.0E-3. Annotations of the two best hits for each contig are given at http://batata.agri.gov.il/ and in Additional file 1. E-value distribution for the top BLAST result for each sequence is given in Figure 3. The E-value distribution of the top hits in the Nr database revealed that 99.5% of the mapped sequences show significant homology (less than 1.0E-5), and 22% of the sequences showed greater than 80% similarity. These results indicated a high level of homology between our sequences and those found in the BLAST database. Similarity distribution of the contigs to their BLAST results is illustrated in Figure 4. Species distribution of the BLAST results is given in Additional file 2, demonstrating that most sweetpotato sequences exhibited similarity to *Vitis vinifera*, *Ricinus communis* and *Populus trichocarpa* sequences (43%, 24% and 18%, respectively), as well as to members of the *Solanaceae* family. Similarity to sequences of *Arabidopsis thaliana* was less than 10%. The relatively low number of hits detected with *Ipomoea batatas* (sweetpotato) may be attributed to the low number of publicly available sequences in the database. The sweetpotato root transcript sequences generated in this study thus add to the recently accumulated sweetpotato sequences [18-21] which can be used for the discovery of new genes involved in root development and functioning and in the initiation of SR formation.

#### ***Functional classification by gene ontology (GO) and by the Kyoto encyclopedia of genes and genomes (KEGG)***

**Figure 5:**
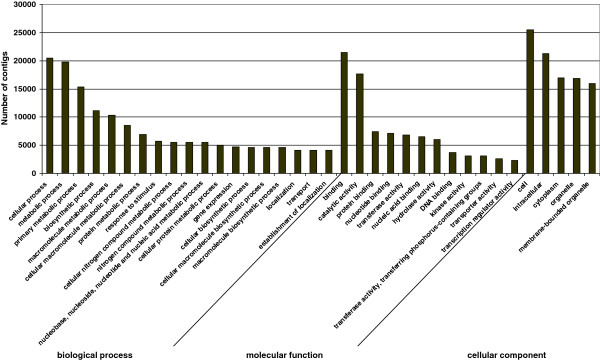
Gene ontology classification of assembled contigs.

To assess whether the sweetpotato root transcriptomic data were indeed representative of roots and SRs, the annotated contigs were assigned to molecular functions using GO. BlastoGO [22] was used to obtain BLAST results for our contigs against the NCBI Nr database and then again to obtain GO annotations for the BLAST results. Ontologizer [23] was used to perform the GO functional classification for the contigs. Of the 40,278 contigs that matched known genes, 34,308 sequences could be grouped into 4,776 different GO categories, and all parental GO terms [i.e. level 2: Biological Process (BP), Molecular Function (MF), Cellular Component (CC)] were assigned (data not shown). Of the GO annotations, 55.3% were associated with BP, 34.5% were associated with MF, and 10.2% were associated with CC (2640, 1648 and 487, respectively). The contigs were further classified using GOSlim [24] and results are presented in Figure 5 and Additional file 3. There was a high representation of cellular process, metabolic process, primary metabolic process, biosynthetic process, macromolecular metabolic process, response to stimulus, localization and transport in the parental category BP, high representation of binding (including nucleotide, protein, carbohydrate and lipid binding), catalytic, transporter and transcription regulator activities in the MF parental category, and high representation of cell, intracellular, cytoplasm, organelle and membrane-bound organelle in the CC category. Similar results have been recently demonstrated for sweetpotato [19-21] and other root transcriptomes, including rice [25], wild rice [26] and *Avena barbata*[27], supporting the quality of our *Georgia Jet* sweetpotato root transcriptome data.

**Figure 6:**
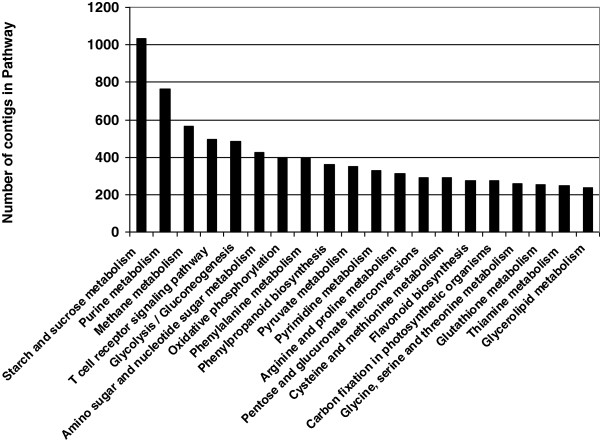
Kyoto Encyclopedia of Genes and Genomes classification of assembled contigs.

To assign the detected contigs to biological pathways, the 55,296 contig sequences were compared using BLASTX with an E-value cutoff of <10E-3 against the KEGG biological pathways database. The contigs were mapped to 140 KEGG pathways, as demonstrated in Additional file 4. Figure 6 summarizes the top 20 represented biological pathways including at least 200 contigs. The highly represented pathways contained starch and sucrose metabolism, purine metabolism, methane metabolism, T-cell receptor signaling pathway, glycolysis, amino sugar and nucleotide sugar metabolism, oxidative phosphorylation, phenylalanine metabolism and phenylpropanoid biosynthesis.

Taken together, the generated database of sweetpotato root transcripts (based upon a mixture of FRs and ISRs) contains genes involved in: (a) classical root functions, such as binding and transport (including transmembrane transport and vesicle-mediated transport) as well as responses to the environment (including response to stimulus), in addition to metabolic processes with high representation of oxidation-reduction processes; (b) metabolic processes and regulation of metabolic processes as well as functions related to development. These results demonstrate the value of the generated transcriptomic data to serve as a database and a reference for continued study aimed at detecting early transcriptome changes in sweetpotato adventitious roots upon initiation of SR formation, as detailed below.

### Early transcriptome changes in sweetpotato adventitious roots upon initiation of SR formation

High-throughput sequencing at the cDNA level, using Illumina GA-IIx technology, was carried out to compare the transcription profiles of two bar-coded samples: (1) a pooled sample of FRs and (2) a pooled sample, of the same age, of ISRs; 17,703,982 and 14,780,229 reads (1 × 100-bp long) were obtained for the FRs and ISRs samples, respectively. The reads were mapped against the set of 55,296 FLX contigs described above, as detailed in ‘Methods’. To enable a direct comparison between the two samples, the read count per EST contig was normalized as described in ‘Methods’. The results of total normalized read counts in the FR and ISR samples are presented in Additional file 5 and are available via our sweetpotato database http://batata.agri.gov.il/. The reads were found to map to 31,284 contigs out of the 55,296 contigs that served as the database, i.e. 56.6% (Additional file 5).

A contig was considered differentially expressed between the ISR and FR samples if two conditions were met: (1) at least 10 reads were mapped to this contig in at least one of the samples and (2) no reads were mapped to the contig in the other sample, or the fold change between read number in each sample was at least 2.5. Thus, a total of 8,353 contigs were found to exhibit differential expression between the two samples. Of these, 4,075 (48.8%) contigs were up-regulated in ISRs compared to FRs and 4,278 (51.2%) contigs were up-regulated in FRs compared to ISRs (Additional file 6); 803 contigs were found to exhibit at least 10-fold higher read number in ISRs compared to FRs and 1,457 contigs were found to exhibit at least 10-fold higher read number in FRs compared to ISR (including contigs with at least 10 mapped reads in one of the samples and no reads in the other sample; Additional file 6 and http://batata.agri.gov.il/).

#### ***Genes involved in sporamin accumulation and carbohydrate metabolism are highly up-regulated during initiation of SR formation***

The list of 70 contigs exhibiting highest differential expression (fold-change) in ISRs compared to FRs is summarized in Table 1. In this list, expression values in the ISR sample, expressed as normalized read number, range between 49 and 4,080 reads per contig and the fold-change in expression compared to the FR sample ranges between 46- and 1,467-fold (Table 1). It should be noted that, for contigs that had no reads in the FR sample, read number was changed from zero to one to enable ‘fold-change’ calculation (Table 1). Highest fold-change expression levels were obtained for contigs representing various members of the sporamin and β-amylase genes, exhibiting 174- to 1,467-fold change in expression. The products of these two genes are known to accumulate to high levels in sweetpotato SRs, with sporamin and β-amylase accounting for about 60% and 5% of total soluble proteins in sweetpotato SRs, respectively [28,29]. Up-regulation of a contig representing an expansin gene in the ISR sample was apparent (contig S_PBL_c3870) exhibiting 98 fold-change in expression. Expansins are known as cell wall-loosening proteins affecting cell expansion in plants and were shown to be involved in root development [[30] and references therein]. In the present work, 15 contigs representing expansin gene sequences were detected by Illumina cDNA sequencing (Additional file 5). Of these, 13 exhibited higher expression in ISRs compared with FRs, including 4 contigs (S_PBL_c1016, S_PBL_c6536, S_PBL_c1824, S_PBL_c6257) that showed high read number (4362, 3066, 1547 and 1446 reads, respectively; Table 1). Our data thus suggest the involvement of expansin in initiation of SR formation and contradict previous findings by Noh et al. [31] showing that down-regulation of the *IbEXP1* expansin gene (exhibiting high homology to contigs S_PBL_c6536 and S_PBL_c1824) in *Ipomoea batatas cv*. *Yulmi* enhanced SR development of sweetpotato.

**Table 1:** Top 70 up-regulated contigs in initiating storage roots compared to fibrous roots

**Contig**	**Length**	**ISR reads**	**FR reads**	**Fold change**	**Annotation**
S_PBL_c2971	936	3135.5	2.1	1466.5	ABB97549.1 Sporamin A precursor [Ipomoea batatas]
S_PBL_c9555	931	1843.7	2.1	862.3	AAA33391.1 Sporamin A precursor
S_PBL_c21625	450	672.6	1.1	629.1	BAA00828.1 Beta-amylase [Ipomoea batatas]
S_PBL_c2075	958	502.3	1.1	469.9	not annotated
S_PBL_c1053	954	455.5	1*	455.6	AAA33390.1 Sporamin B
S_PBL_c54	918	2525.6	6.4	393.8	P10917.1 Sporamin A; Flags: Precursor
S_PBL_c17697	844	753.0	2.1	352.2	AAB52547.1 Sporamin precursor
S_PBL_c18468	263	311.5	1*	311.5	not annotated
S_PBL_c15531	438	2823.1	11.8	240.1	AAA33391.1 Sporamin A precursor
S_PBL_c8154	557	238.5	1*	238.5	not annotated
S_PBL_c3494	967	252.6	1.1	236.3	AAL55800.1 Sporamin [Ipomoea batatas]
S_PBL_c3628	759	195.5	1*	195.5	ABB90968.1 Sporamin B precursor [Ipomoea batatas]
S_PBL_c12997	481	190.8	1*	190.8	not annotated
S_PBL_c2027	1665	186.1	1.1	174.1	BAA02286.1 Beta-amylase [Ipomoea batatas]
S_PBL_lrc44407	487	158.1	1*	158.1	not annotated
S_PBL_c25030	652	150.6	1*	150.6	XP_001677106.1 Hypothetical protein CBG16734 [Caenorhabditis briggsae AF16]
S_PBL_c12797	567	134.7	1*	134.7	not annotated
S_PBL_c21769	669	131.0	1*	131.0	not annotated
S_PBL_lrc53020	508	386.3	3.2	120.5	not annotated
S_PBL_lrc25060	898	128.2	1.1	119.9	CAO23244.1 unnamed protein product [Vitis vinifera]
S_PBL_c34561	465	117.9	1*	117.9	XP_002298721.1 predicted protein [Populus trichocarpa]
S_PBL_c9857	630	581.8	5.3	108.9	not annotated
S_PBL_c3428	1350	577.2	5.3	108.0	not annotated
S_PBL_c18337	561	1035.5	9.6	107.6	AAX27911.1 unknown [Schistosoma japonicum]
S_PBL_c3870	696	104.8	1.1	98.0	ABB83474.1 Beta-expansin precursor [Solanum lycopersicum]
S_PBL_c45358	560	96.3	1*	96.3	CAO47717.1 unnamed protein product [Vitis vinifera]
S_PBL_c4363	735	199.2	2.1	93.2	not annotated
S_PBL_c20072	900	91.7	1*	91.7	P10965.1 Sporamin B; Flags: Precursor
S_PBL_c3830	725	291.8	3.2	91.0	EEF50674.1 conserved hypothetical protein [Ricinus communis]
S_PBL_c7863	397	90.7	1*	90.7	not annotated
S_PBL_lrc52376	494	189.9	2.1	88.8	not annotated
S_PBL_c2723	895	93.5	1.1	87.5	CAO43166.1 unnamed protein product [Vitis vinifera]
S_PBL_c1850	702	182.4	2.1	85.3	not annotated
S_PBL_c15391	502	89.8	1.1	84.0	not annotated
S_PBL_c26497	974	83.3	1*	83.3	XP_002327563.1 predicted protein [Populus trichocarpa]
S_PBL_c48117	388	262.9	3.2	82.0	P10933.1 Ferredoxin-NADP reductase, leaf isozyme, chloroplastic
S_PBL_c20358	683	85.1	1.1	79.6	CAO22863.1 unnamed protein product [Vitis vinifera]
S_PBL_c3576	454	169.3	2.1	79.2	not annotated
S_PBL_c8690	813	75.8	1*	75.8	AAU93595.1 HVA22-like protein c, putative [Solanum demissum]
S_PBL_c7526	860	79.5	1.1	74.4	not annotated
S_PBL_lrc53818	757	4080.3	58.8	69.4	BAF47746.1 ADP-glucose pyrophosphorylase beta subunit IbAGPb1A [Ipomoea batatas]
S_PBL_lrc52525	509	73.0	1.1	68.3	XP_002274272.1 PREDICTED: hypothetical protein [Vitis vinifera]
S_PBL_lrc30491	523	145.9	2.1	68.3	P27598.1 Starch phosphorylase L
S_PBL_c41294	520	68.3	1.1	63.9	not annotated
S_PBL_c32227	418	63.6	1*	63.6	not annotated
S_PBL_c54084	609	135.6	2.1	63.4	not annotated
S_PBL_c31475	869	66.4	1.1	62.1	not annotated
S_PBL_lrc52682	527	66.4	1.1	62.1	XP_002306992.1 predicted protein [Populus trichocarpa]
S_PBL_c10411	496	64.5	1.1	60.4	BAA31699.1 PKn2 [Ipomoea nil]
S_PBL_lrc27049	954	255.4	4.3	59.7	not annotated
S_PBL_c14744	705	63.6	1.1	59.5	EEF31509.1 Ubiquitin-protein ligase bre-1, putative [Ricinus communis]
S_PBL_c18129	413	1393.8	23.5	59.3	BAF47744.2 ADP-glucose pyrophosphorylase alpha subunit IbAGPa1 [Ipomoea batatas]
S_PBL_c7810	531	123.5	2.1	57.8	not annotated
S_PBL_c14132	307	57.1	1*	57.1	not annotated
S_PBL_c1234	886	2045.8	36.3	56.3	BAF47746.1 ADP-glucose pyrophosphorylase beta subunit IbAGPb1A [Ipomoea batatas]
S_PBL_c695	596	721.2	12.8	56.2	P27598.1 Starch phosphorylase L
S_PBL_c30129	726	56.1	1*	56.1	AAP41026.1 NTA15 protein [Nicotiana tabacum]
S_PBL_c12308	666	115.1	2.1	53.8	P93262.1 Phosphoglucomutase, cytoplasmic
S_PBL_c28076	644	53.3	1*	53.3	CAO21883.1 unnamed protein product [Vitis vinifera]
S_PBL_c16839	282	55.2	1.1	51.6	not annotated
S_PBL_c47419	537	55.2	1.1	51.6	EEF50087.1 Serine-threonine protein kinase, plant-type, putative [Ricinus communis]
S_PBL_c51126	492	661.3	12.8	51.6	BAF47744.2 ADP-glucose pyrophosphorylase alpha subunit IbAGPa1 [Ipomoea batatas]
S_PBL_c19033	878	51.4	1*	51.4	not annotated
S_PBL_lrc28526	555	51.4	1*	51.4	not annotated
S_PBL_c7145	689	161.8	3.2	50.5	not annotated
S_PBL_c12588	493	49.6	1*	49.6	not annotated
S_PBL_lrc53425	456	211.4	4.3	49.4	BAF47746.1 ADP-glucose pyrophosphorylase beta subunit IbAGPb1A [Ipomoea batatas]
S_PBL_c21020	403	103.8	2.1	48.6	not annotated
S_PBL_c1397	989	408.8	8.6	47.8	BAF47744.2 ADP-glucose pyrophosphorylase alpha subunit IbAGPa1 [Ipomoea batatas]
S_PBL_c19766	429	984.1	21.4	46.0	P27598.1 Starch phosphorylase L

Expression values are expressed as normalized read number. ‘Fold change’ is calculated as ISR reads/FR reads. One annotation is given for each contig. Annotations of the two best hits for each contig (E value ≤ 1.0E-3) are available in Additional file 1. *ISR,* Initiating storage root, *FR* fibrous root. * - Read counts were changed from zero to one to enable ‘Fold change’ calculation (avoid dividing by zero).

In addition, included in this list are genes involved in starch biosynthesis, coding for α and β subunits of ADP glucose pyrophosphorylase (AGPase; exhibiting high expression levels: 4,080 and 1,394 reads for specific β and α members, respectively) and phosphoglucomutase. This list includes a significant number of contigs without annotation (30 contigs; 43%). Looking into the expression levels (number of Illumina-generated reads) of additional starch biosynthesis genes not included in Table 1, high expression of contigs representing several members of granule-bound starch synthase was detected (in the range of 498 to 4,757 reads, exhibiting 15- to 23-fold higher expression in ISRs compared to FRs, for contigs S_PBL_c3042, S_PBL_c9881, S_PBL_c4145; Additional file 5 and http://batata.agri.gov.il/). The high expression level (83-fold higher in ISR compared to FR) of a contig exhibiting homology to ferredoxin suggests its involvement in the redox modulation and activation of AGPase [32].

**Figure 7:**
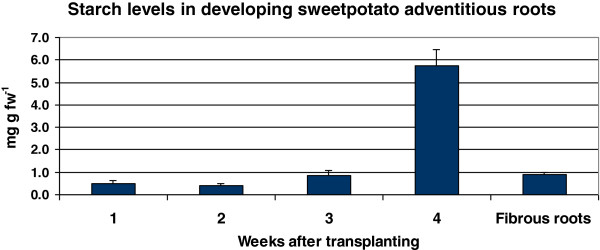
**Starch levels in developing adventitious roots around the timing of storage root initiation.** Root samples of *Georgia Jet* were pooled from five to seven plants at 1, 2, 3 and 4 weeks after transplanting, spanning the period of storage root (SR) initiation. Data represent an average of three biological replicates ± SE. “Fibrous roots” represent fibrous root samples derived from plants at 8 weeks after transplanting (at least 4 weeks past the period of SR initiation).

To follow changes in starch levels in the roots of *Georgia Jet* during the first 4 weeks after transplanting, spanning the timing of SR initiation [7], we evaluated starch concentrations in the root system at several time points after transplanting (Figure 7). Samples were taken from the entire root system, since up to 3 weeks after transplanting SR initiation could not be distinguished by microscopic analysis of root cross sections. The data indicated a peak of starch accumulation (an over fivefold increase) at the initiation of SR formation, between 3 and 4 weeks after transplanting (Figure 7). Starch levels of non-initiated, 2-month-old FRs were found to be relatively low (0.9 mg g fw^-1^; Figure 7), suggesting that the increased starch in the pooled root sample was contributed by the ISRs. These data are in agreement with the elevated expression of genes involved in starch biosynthesis detected by our Illumina sequencing (Table 1). To the best of our knowledge, the accumulated results represent novel data with respect to early elevation in expression of starch biosynthesis genes as well as starch accumulation, marking the initial phase of SR development.

#### ***Regulators of meristematic tissue identity and maintenance, as well as of cell division, are up-regulated in ISRs***

A contig representing a class I knotted1-like protein (S_PBL_c10411) homologous to *Ipomoea nil PKn2* (knotted-like gene) mRNA (AB016000.1) and *Ibkn4* of *Ipomoea batatas* (AB016000.1; class-I knotted1-like) showed 60-fold higher expression in ISRs compared to FRs (Table 1). Knotted1-like homeobox (KNOX) transcription factors are regulators involved in the establishment and maintenance of plant meristems, such as the shoot apical meristem [[33] and references therein]. The cambium is a stem-like tissue [34]; the term cambium refers to one or several layers of initials, analogous to the stem cells proposed for other meristems [[35] and references therein]. Divisions of these initials then produce phloem or xylem mother cells, which in turn can undergo several rounds of cell division before differentiating [35]. Schrader et al. [35] identified molecular markers that characterize the cambial zone in poplar, including genes that regulate meristem identity and mark the cambium initials, and genes that control cell division and mark xylem mother cells. Of these genes, *KNOX* genes such as the poplar *PttKNOX1*, *PttKNOX2* and *PttKNOX6* showed high expression in cambial samples [35].

As indicated previously by Wilson and Lowe [5], and as demonstrated by us [7] for both the leading Israeli sweetpotato variety *Georgia Jet* and an important USA variety, *Beauregard*, initiation of SR formation is marked by the development of AC cells adjacent to xylem elements, starting 3 to 4 weeks after transplanting. Repeated division of these cambium cells leads to the formation of rows of thin-walled parenchyma cells that will form the storage tissue of the SR [7]. Several contigs homologous to *KNOX* genes were detected by us in the sweetpotato root transcriptome, with specific members exhibiting higher expression in ISRs compared to FRs (Table 2). It is interesting to note that all identified members of the class I knotted1-like proteins exhibited at least twofold higher expression in ISRs compared to FRs, while genes belonging to the class II knotted1-like family exhibited a versatile expression pattern (Table 2). *KNOXI* genes have been previously shown to be involved in the development of sweetpotato SRs [17,36], regulating cytokinin levels in that organ. Tanaka et al. [17] identified three different *KNOXI* gene fragments—*ibkn1*, *ibkn2* and *ibkn3*—in sweetpotato SR. Phylogenetic analysis of putative amino acid sequences showed that *ibkn1* is homologous to the SHOOT MERISTEMLESS (STM) gene of *Arabidopsis thaliana*, while *ibkn2* and *ibkn3* are homologous to the BP gene. Expressions of *ibkn1*, *ibkn2* and *ibkn3* were faint or undetectable in fibrous, non-storage roots [17]. Mele eta al. [37] suggested in Arabidopsis that the BP gene regulates the lignin pathway, thus repressing premature cell differentiation. The class I knotted1-like genes found by us to be up-regulated in ISRs included *ibkn2* and *ibkn3* homologues as well as additional family members (Table 2).

**Table 2:** Expression, in initiating storage roots and fibrous roots, of genes involved in meristem- and cell-division regulation

**Contig**	**Length**	**ISR reads**	**FR reads**	**Annotation**
**Meristem regulation**				
S_PBL_10411	496.0	64.5	1.1	BAA31699.1 PKn2 [Ipomoea nil]
S_PBL_c8137	854.0	29.0	2.1	BAF93479.1 Class-I knotted1-like homeobox protein IBKN2 [Ipomoea batatas]
S_PBL_c9098	625.0	52.4	25.7	AAO33774.1 Knotted protein TKN4 [Lycopersicon esculentum] Class I knox
S_PBL_c11762	744.0	171.2	16.0	BAF93480.1 Class-I knotted1-like homeobox protein IBKN3 [Ipomoea batatas]
S_PBL_c31412	461.0	97.3	11.8	BAF93480.1 Class-I knotted1-like homeobox protein IBKN3 [Ipomoea batatas]
S_PBL_lrc34271	455.0	2.8	44.0	BO33481.1 Class II KNOX homeobox transcription factor [Medicago truncatula]
S_PBL_c5315	807.0	30.9	25.7	BAF95776.1 Class 2 knotted1-like protein [Nicotiana tabacum]
S_PBL_c6714	747.0	6.5	3.2	ABH03531.1 Class II knotted-like homeobox protein [Prunus persica]
S_PBL_c12444	767.0	644.5	809.3	NP_197904.1 KNAT3 [Arabidopsis thaliana] Class II KNOTTED1-like homeobox gene 3
**Cell division regulation**				
S_PBL_c25867	444.0	18.7	0.0	BAA20410.1 A-type cyclin [Catharanthus roseus]
S_PBL_c32270	712.0	42.1	5.3	AAV41032.1 Cyclin D-like protein [Nicotiana tabacum]
S_PBL_c29324	408.0	213.3	336.7	EEF32676.1 Cyclin-L2, putative [Ricinus communis]
S_PBL_c3050	747.0	141.2	100.5	AAR01224.1 Cyclin T1 [Medicago truncatula]
S_PBL_c1272	1042.0	530.4	108.0	BAE06268.1 Cyclin-dependent kinase A1 [Scutellaria baicalensis]
S_PBL_c1655	489.0	44.9	3.2	EEF38677.1 Cyclin-dependent kinases regulatory subunit, putative [Ricinus communis]
S_PBL_c33143	467.0	35.5	6.4	AAG01532.1 Cyclin-dependent kinase B1-1 [Nicotiana tabacum]
S_PBL_c27090	768.0	112.3	54.5	CAC51391.1 Cyclin dependent kinase C [Solanum lycopersicum]
S_PBL_c28094	482.0	81.4	11.8	ABN58480.1 Cyclin-dependent kinase [Actinidia chinensis]

Expression is given as the normalized number of reads derived from the Illumina sequencing data as detailed in ‘Methods’. *ISR*, Initiating storage root, *FR*, Fibrous root.

Almost all of the cell-division-regulating genes that were detected in this work showed higher expression in ISRs compared to FRs, including genes encoding cyclin A-like and cyclin D-like proteins and five cyclin-dependent kinases (Table 2 and Additional file 5). These results are in agreement with the observed increase in the number of AC cells in sweetpotato ISR tissue sections (Figure 1) and with reports of accelerated cell division upon SR initiation in other sweetpotato varieties [36]. These results are novel and additional work is needed to characterize the spatial expression of these genes in root sections at different time points during and following SR initiation.

#### ***Genes down-regulated during initiation of SR formation***

To identify root functions and processes that are down-regulated during the development of FRs into SRs, we looked into genes represented by contigs that exhibit significantly higher fold-change expression in FRs compared to ISRs. The list of 70 contigs exhibiting highest differential expression in FRs compared to ISRs is summarized in Table 3. This list contains a relatively large number of non-annotated contigs, in addition to contigs that represent genes involved in root development and function as well as defense, such as a metallothionein-like protein which has been shown in rice to be involved in root formation [38] and was found to exhibit a high read number (Table 3). Metallothioneins are cysteine-rich metal-binding proteins of low molecular mass that are mainly involved in maintaining metal homeostasis, metal detoxification and stress/defense responses [38]. Additional genes known to be involved in stress response (including to drought and salt stresses) and found to exhibit down-regulated expression in ISRs compared to FRs included an osmotin-like protein (a member of the class 5 pathogenesis-related proteins) and pathogenesis-related protein PR10a (Table 3; [39]), as well as a cysteine protease shown to be involved in senescence and the osmotic stress response [40].

**Table 3:** Top 70 up-regulated contigs in fibrous roots compared to initiating storage roots

**Contig**	**Length**	**ISR reads**	**FR reads**	**Fold change**	**Annotation**
S_PBL_c4848	510	0.9	546.3	607.0	not annotated
S_PBL_c22976	665	2.8	756.9	270.3	XP_002327317.1 Predicted protein [Populus trichocarpa]
S_PBL_lrc52198	626	0.9	228.8	254.2	AAK27968.1 Cysteine protease [Ipomoea batatas]
S_PBL_c1692	703	1*	251.2	251.2	not annotated
S_PBL_c14075	626	1*	232	232.0	XP_001837463.1 Tubulin alpha chain [Coprinopsis cinerea okayama7#130]
S_PBL_c36764	703	3.7	856.3	231.4	XP_002303479.1 predicted protein [Populus trichocarpa]
S_PBL_c1452	539	0.9	206.3	229.2	AAK27968.1 Cysteine protease [Ipomoea batatas]
S_PBL_c25084	735	1*	209.5	209.5	CAO14793.1 unnamed protein product [Vitis vinifera]
S_PBL_c2976	600	26.2	5316.4	202.9	Q40157.1 Metallothionein-like protein type 2 A
S_PBL_lrc52159	665	0.9	174.3	193.7	not annotated
S_PBL_c43111	446	0.9	168.9	187.7	not annotated
S_PBL_c39992	480	1*	187.1	187.1	XP_001874004.1 predicted protein [Laccaria bicolor]
S_PBL_c18994	719	20.6	3790.8	184.0	not annotated
S_PBL_c28403	834	0.9	164.6	182.9	XP_001884841.1 Delta 9-fatty acid desaturase protein [Laccaria bicolor]
S_PBL_c30965	742	0.9	153.9	171.0	CAO23367.1 unnamed protein product [Vitis vinifera]
S_PBL_c29652	772	0.9	151.8	168.7	XP_002304020.1 predicted protein [Populus trichocarpa]
S_PBL_c12455	525	34.6	5763.2	166.6	Q40157.1 Metallothionein-like protein type 2 A
S_PBL_c5087	783	2.8	465	166.1	not annotated
S_PBL_c18075	609	4.7	767.6	163.3	not annotated
S_PBL_c44512	388	0.9	145.4	161.6	XP_002279608.1 hypothetical protein [Vitis vinifera]
S_PBL_c19899	443	0.9	144.3	160.3	not annotated
S_PBL_c14724	532	1*	156.1	156.1	CAN80314.1 hypothetical protein [Vitis vinifera]
S_PBL_c30195	672	0.9	136.8	152.0	not annotated
S_PBL_c8847	687	1*	150.7	150.7	XP_002330606.1 predicted protein [Populus trichocarpa]
S_PBL_c6142	645	1.9	275.8	145.2	XP_002263033 Peroxidase 10-like [Vitis vinifera]
S_PBL_c11711	510	7.5	1082.9	144.4	XP_001880208.1 Fumarate reductase [Laccaria bicolor]
S_PBL_c35569	653	3.7	533.5	144.2	not annotated
S_PBL_c337	838	27.1	3771.6	139.2	AAL79832.2 Osmotin-like protein [Solanum nigrum]
S_PBL_c50215	567	1.9	261.9	137.8	not annotated
S_PBL_lrc32508	540	0.9	124	137.8	not annotated
S_PBL_c12800	554	1.9	260.8	137.3	XP_001881080.1 Histone 2A [Laccaria bicolor]
S_PBL_c13942	648	3.7	504.6	136.4	XP_001888404.1 Sphingolipid C9-methyltransferase [Laccaria bicolor]
S_PBL_c837	483	1.9	258.7	136.2	not annotated
S_PBL_c14357	689	3.7	488.6	132.1	EAY93058.1 hypothetical protein OsI_14861 [Oryza sativa Indica Group]
S_PBL_c44050	575	1.9	242.7	127.7	XP_001834214.1 hypothetical protein CC1G_09714 [Coprinopsis cinerea okayama7#130]
S_PBL_c47284	674	0.9	114.4	127.1	EEF39207.1 Rhicadhesin receptor precursor, putative [Ricinus communis]
S_PBL_c29183	616	0.9	113.3	125.9	not annotated
S_PBL_c15554	601	13.1	1644.2	125.5	Q40157.1 Metallothionein-like protein type 2 A
S_PBL_c28805	440	1*	122.9	122.9	CAO44491.1 unnamed protein product [Vitis vinifera]
S_PBL_c9181	557	1.9	230.9	121.5	AAY87888.1 Glutathione peroxidase [Taiwanofungus camphoratus]
S_PBL_lrc53720	379	52.4	6313.8	120.5	not annotated
S_PBL_c18857	626	1*	117.6	117.6	AAK27968.1 Cysteine protease [Ipomoea batatas]
S_PBL_c5350	526	1.9	221.3	116.5	not annotated
S_PBL_c41001	434	0.9	103.7	115.2	XP_572855.1 Polysaccharide synthase [Cryptococcus neoformans]
S_PBL_c17858	525	1*	113.3	113.3	not annotated
S_PBL_c30000	488	0.9	101.6	112.9	XP_001838960.1 hypothetical protein CC1G_05513 [Coprinopsis cinerea okayama7#130]
S_PBL_c25576	582	0.9	100.5	111.7	not annotated
S_PBL_c6293	718	12.2	1345.9	110.3	not annotated
S_PBL_c37132	470	0.9	95.1	105.7	not annotated
S_PBL_c3863	605	1.9	198.8	104.6	CAN80314.1 hypothetical protein [Vitis vinifera]
S_PBL_c12972	545	15.9	1651.7	103.9	AAL16409.1 Pathogenesis-related protein PR10a [Nicotiana tabacum]
S_PBL_c2399	892	2.8	290.8	103.9	CAO22665.1 unnamed protein product [Vitis vinifera]
S_PBL_c7735	565	1.9	196.7	103.5	NP_200248.1 Late embryogenesis abundant protein-related [Arabidopsis thaliana]
S_PBL_c28770	429	1.9	195.6	102.9	not annotated
S_PBL_c33041	444	1.9	194.6	102.4	not annotated
S_PBL_c27326	782	6.5	664.9	102.3	XP_001890326.1 GPA1 heterotrimeric G-protein alpha subunit [Laccaria bicolor]
S_PBL_c18989	540	1.9	193.5	101.8	AAK27968.1 Cysteine protease [Ipomoea batatas]
S_PBL_c6361	637	8.4	855.2	101.8	XP_001880208.1 Fumarate reductase [Laccaria bicolor]
S_PBL_c15414	674	1*	100.5	100.5	AAL14199.1 Cysteine proteinase precursor [Ipomoea batatas]
S_PBL_c15414	674	1*	100.5	100.5	AAL14199.1 Cysteine proteinase precursor [Ipomoea batatas]
S_PBL_c18957	652	1.9	190.3	100.2	not annotated
S_PBL_c12342	570	1*	98.4	98.4	not annotated
S_PBL_c10766	501	1*	94.1	94.1	not annotated
S_PBL_c40440	403	1*	94.1	94.1	not annotated
S_PBL_c11884	622	1*	93	93	XP_002273655.1 PREDICTED: hypothetical protein [Vitis vinifera]
S_PBL_lrc28732	494	1*	87.7	87.7	not annotated
S_PBL_c24730	463	1*	86.6	86.6	not annotated
S_PBL_c32483	699	1*	86.6	86.6	not annotated
S_PBL_c14615	660	1*	84.5	84.5	EEF31626.1 Kiwellin, putative [Ricinus communis]
S_PBL_c28823	729	1*	83.4	83.4	XP_001831241.1 hypothetical protein CC1G_00788 [Coprinopsis cinerea okayama7#130]

Expression values are expressed as normalized read number. ‘Fold change’ is calculated as FR reads/ISR reads. One annotation is given for each contig. Annotations of the two best hits for each contig (E value ≤ 1.0E-3) are available in Additional file 1. *ISR*, Initiating storage root, *FR*, Fibrous root. * - Read counts were changed from zero to one to enable ‘Fold change’ calculation.

In addition, contig S_PBL_c6142, which showed an over 100-fold higher read number in FRs compared with SRs, was found to exhibit significant homology to peroxidase 10-like mRNA (EC 1.11.1.7) of *Vitis vinifera* (3E-81) and *Ricinus communis* (2E-79), involved in the phenylpropanoid and lignin biosynthesis pathways. Angiosperm lignins are complex phenolic polymers that consist mostly of guaiacyl and syringyl units, together with small or trace amounts of *p* hydroxyphenyl units. Monolignols are synthesized in the cytosol and transported to the cell wall, where their oxidation generates lignins [41]. From a functional point of view, lignins impart strength to cell walls, facilitate water transport, and impede the degradation of wall polysaccharides, thus acting as a major line of defense against pathogens, insects, and other herbivores [42]. In sweetpotato, Togari [4] proposed a direct link between lignification and SR initiation, suggesting that lignification inhibits SR development. The relationship between stele lignification and inability of adventitious roots to develop into SRs has also been observed by Wilson and Lowe [5], Belehu et al. [6] and Villordon et al. [7]. Togari [4] suggested that genetic and environmental factors influence the balance between cambium development and lignification, which in turn determines to a large degree the final SR yield. Indeed, looking into the expression levels of contigs representing additional lignin biosynthesis genes not included in Table 3, such as S_PBL_c158, S_PBL_c20480 and S_PBL_lrc53688, representing coumaroyl-CoA synthase, caffeoyl-CoA O-methyltransferase and cinnamyl alcohol dehydrogenase, respectively, more than sevenfold reduced expression in ISRs compared to FRs was detected (Additional files 5 and 6). This gene-expression pattern parallels the reduced lignification observed in tissue sections of ISRs compared to FRs (Figure 1; [7] and data not shown). In this context of potential cross talk between establishment and development of the cambium meristem on the one hand, and lignification on the other, it is interesting to note that data from studies on a specific peach class I knotted-like gene (*knope1*) suggest that KNOPE1 prevents cell lignification by repressing lignin genes during peach stem primary growth [43].

#### ***Validation of differential expression between ISRs and FRs revealed by the use of Illumina-based sequencing at the cDNA level***

**Figure 8:**
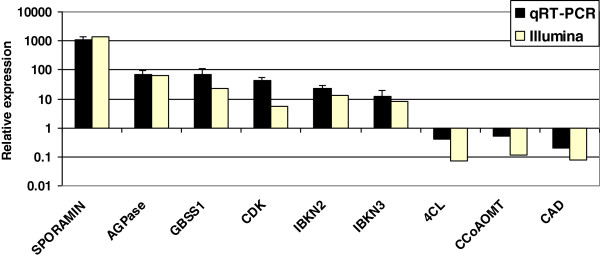
**Validation by quantitative on-line RT-PCR analyses of the differential expression between sweetpotato initiating storage and fibrous roots revealed by the use of Illumina-based sequencing at the cDNA level.** Expression levels were measured in initiating storage root (ISR) and fibrous root (FR) samples of *Georgia Jet* sweetpotato variety, using at least four biological replicates. Each replicate consisted of cDNA representing pooled root tissue from 30 plants. Quantitative RT-PCR was performed and values were normalized relative to the expression levels of 18S rRNA in the same cDNA sample. Expression data are the means (± SE) of at least four replicates and are presented as relative expression values of the respective gene in the ISR sample relative to its expression in the FR sample (ISR/FR). The Y axis has a logarithmic scale. ADP glucose pyrophosphorylase – AGPase; coumaroyl-CoA synthase – 4CL; caffeoyl-CoA O-methyltransferase – CCoAOMT; cinnamyl alcohol dehydrogenase – CAD.

The validity of the expression differences detected by Illumina-based sequencing was examined at the RNA level, using on-line quantitative (q) RT-PCR. Results for nine selected contigs are presented (Figure 8), representing five groups of genes that were found to be differentially expressed by the Illumina results: a gene encoding sporamin, a sweetpotato storage protein accounting for about 60% of total soluble proteins, genes encoding enzymes involved in starch biosynthesis (AGPase and granule-bound starch synthase 1—GBBS1), genes regulating meristem identity (homologous to class-I knotted-like homeobox proteins Ibkn2 and Ibkn3), a gene involved in cell division regulation (cyclin-dependent kinase—CDK) and genes encoding enzymes of lignin biosynthesis (4 coumaroyl-CoA synthase 1—4CL; caffeoyl-CoA O-methyltransferase 2—CCoAOMT; cinnamyl alcohol dehydrogenase—CAD). Results are presented as relative expression values of the respective genes in the ISR sample relative to their expression in the FR sample. In all cases tested, the qRT-PCR analyses confirmed the results of the Illumina analyses (Figure 8), namely elevated expression of gene sequences involved in starch biosynthesis and sporamin accumulation, of the gene encoding CDK and of genes belonging to the class-I knotted-like homeobox protein family, and down-regulation of genes encoding enzymes of lignin biosynthesis in ISRs. However, some of the relative expression values differed between the two expression analysis methods. For example, relative expression values detected by the Illumina and qRT-PCR methods for CDK, 4CL and CAD were 5.6, 0.07, 0.08 and 44.8, 0.4 and 0.2, respectively. It should be noted that the Illumina-based expression results were derived from one biological replicate of the ISR sample and one biological replicate of the FR sample (each sample consisting of cDNA representing pooled root tissue of 30 plants), while the qRT-PCR results were derived from at least four biological replicates (each sample consisting of cDNA representing pooled root tissue of 30 plants). Overall, the qRT-PCR results validated the Illumina-based cDNA sequencing results, allowing for the interpretation of global gene expression trends. Independent validations are still required to accurately measure the expression of each gene of interest.

#### ***Analysis of over-represented GO terms in the subset of differentially expressed contigs, in either ISRs or FRs, relative to the root transcriptome database***

**Figure 9:**
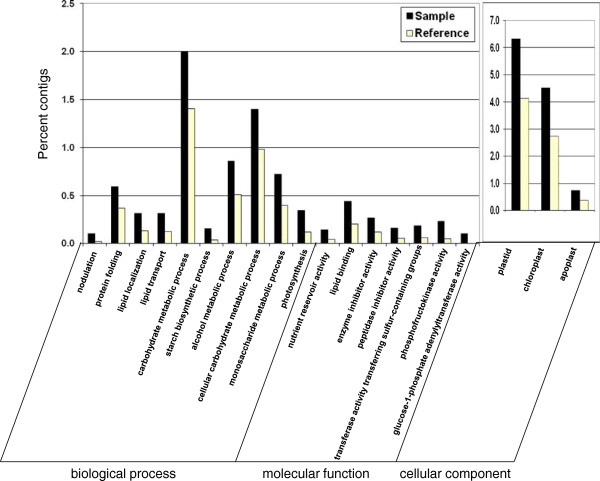
**GO-term enrichment in the initiating storage root sample (‘sample’) relative to the root transcriptome database (‘reference’).** Initiating storage root sample histograms are indicated in black, while the ‘reference’ histograms are indicated in yellow.

The differentially expressed contigs in ISRs and FRs were analyzed for GO-category enrichment relative to the root transcriptome database using AgriGO [44]. The results for the up-regulated contigs in ISRs are presented in Figure 9 and Additional files 7 and 8. The up-regulated contigs in ISRs were found to contain 21 significantly enriched (FDR ≤ 0.05) GO functional terms in the biology process category, including three ‘level 3’ and four ‘level 4’ terms (“carbohydrate metabolic process”, GO:0005975; “alcohol metabolic process”, GO:0006066; “lipid localization”, GO:0010876 as well as “photosynthesis”, GO:0015979; “lipid transport”, GO:0006869; “cellular carbohydrate metabolic process”, GO:0044262; “nodulation”, GO:0009877, respectively). The highly enriched (FDR = 0.0003) “carbohydrate metabolic process” term included several contigs of starch phosphorylase, phosphoglucomutase, starch branching enzyme and 1,3-β-glucosidase, and its daughter terms included “monosaccharide metabolic process” (GO:0005996) and “starch biosynthetic process” (GO:0019252; including several contigs of ADP glucose pyrophosphorylase and starch synthase). The results thus point to enrichment in ISRs of carbohydrate metabolism and starch biosynthesis functions, in accordance with the biochemical analysis results indicating starch accumulation at the time of SR initiation (Figure 7). An additional and interesting enriched term was “protein folding” (GO:0006457), a ‘level 7’ term that included contigs of cyclophilin, peptidyl-prolyl cis/trans isomerase, calnexin CONSTANS interacting protein 3, heat-shock protein and chaperones, and was connected to the term “protein thiol-disulfide exchange” (GO:0006467).

As for molecular function, the up-regulated contigs belonged to 13 significantly enriched GO terms, including the ‘level 1’ term “nutrient reservoir activity” (GO:0045735), containing several contigs of sporamin A and sporamin B precursors, the ‘level 2’ terms “lipid binding” (GO:0008289), “enzyme inhibitor activity” (GO:0004857), and the ‘level 3’ terms “transferase activity, transferring sulfur-containing groups” (GO:0016782) and “peptidase inhibitor activity” (GO:0030414). The most significantly enriched term was the ‘level 6’ term “phosphofructokinase activity” (GO:0008443). “Glucose-1-phosphate adenylyltransferase (GO:0008878), containing several contigs of ADP-glucose pyrophosphorylase subunits α and β, was also significantly enriched (FDR = 0.028). The GO terms of “plastid” (GO:0009536) and “chloroplast” (GO:0009507) and their daughter terms were the most highly enriched in the cellular component category (with FDR values of 4.03E-26 and 4.02E-25, respectively), in accordance with involvement of amyloplasts in SR starch accumulation. The “apoplast” term (GO:0048046) was also found to be significantly enriched in the ISR sample (Figure 9).

**Figure 10:**
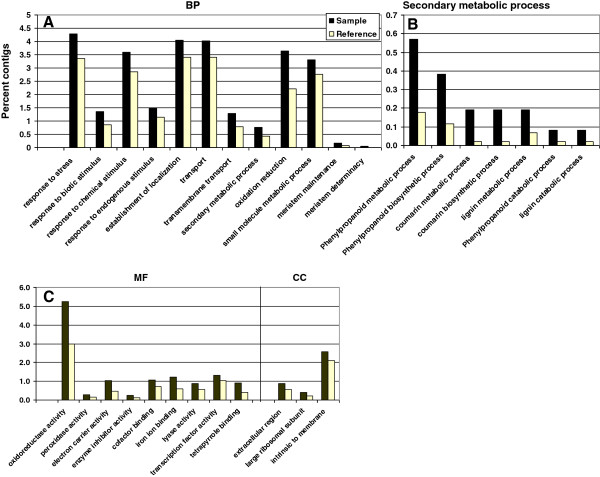
**GO-term enrichment in the fibrous root sample (‘sample’) relative to the root transcriptome database (‘reference’).** Fibrous root sample histograms are indicated in black, while the ‘reference’ histograms are indicated in yellow. **A**. Biological process (BP). **B**. GO terms included in the ‘Secondary metabolic process’ BP category. **C**. Molecular function (MF) and cellular component (CC) categories.

The up-regulated contigs in FRs were found to represent a larger number of different enriched GO functional categories (FDR ≤ 0.05) compared to ISRs (Figure 10 and Additional files 9 and 10). Among the enriched terms represented by the up-regulated contigs in FRs, a total of 74 terms were included in the BP category, containing the ‘level 2’ terms “oxidation reduction” (GO:0055114), “small molecule metabolic process” (GO:0044281), “response to stress” (GO:0006950), “response to biotic stimulus” (GO:0009607), “response to chemical stimulus” (GO:0042221) and “secondary metabolic process” (GO:0019748), and the ‘level 3’ terms “response to endogenous stimulus” (GO: GO:0009719) and “transport” (GO:0006810), which in turn included “transmembrane transport” (GO:0055085). The enriched “secondary metabolic process” term exhibited high enrichment in “phenylpropanoid metabolic process” (GO:0009698; containing several contigs of coumaroyl CoA synthase and phenylalanine ammonia lyase), “phenylpropanoid biosynthetic process” (GO:0009699), “coumarin metabolic process” (GO:0009804), “coumarin biosynthetic process” (GO:0009805; containing contigs of p-coumaroyl quinate/shikimate 3′-hydroxylase, caffeoyl coenzyme A 3-o-methtyl transferase), “lignin metabolic process” (GO:0009808), “phenylpropanoid catabolic process” (GO:0046271 see Figure 10) and “lignin catabolic process” (GO:0046274). An interesting group of enriched functional terms (FDR ≤ 0.032) included: “meristem determinacy” (GO:0010022), “meristem maintenance” (GO:0010073) and “inflorescence meristem growth” (GO:0010450), which contained several contigs representing at least three ultrapetala 1-like proteins. Ultrapetala 1-like protein is a putative transcription factor that acts as a key negative regulator of cell accumulation in *Arabidopsis* shoot and floral meristems [45]. The higher-plant shoot apical meristem is a dynamic structure that continuously produces cells which become incorporated into new leaves, stems and flowers. The maintenance of a constant flow of cells through the meristem depends on coordination of two antagonistic processes: self-renewal of the stem cell population and initiation of the lateral organs. This coordination is stringently controlled by gene networks that contain both positive and negative components. Carles et al. [45,46] defined the ULTRAPETALA1 (*ULT1*) gene as a key negative regulator of cell accumulation in *Arabidopsis* shoot and floral meristems, because mutations in *ULT1* caused enlargement of inflorescence and floral meristems, the production of supernumerary flowers and floral organs, and a delay in floral meristem termination.

In the MF category, the up-regulated contigs belonged to 78 significantly enriched GO terms, including electron carrier, transporter, antioxidant and catalytic activities, and binding. Catalytic activity included “oxidoreductase activity” (GO:0016491), which in turn included “oxidoreductase activity, acting on diphenols and related substances as donors, oxygen as acceptor” (GO:0016682), “oxidoreductase activity, acting on paired donors, with incorporation or reduction of molecular oxygen, NADH or NADPH as one donor, and incorporation of one atom of oxygen” (GO:0016709) and “trans-cinnamate 4 monooxygenase activity” (GO:0016710), as well as “oxidoreductase activity, acting on paired donors, with incorporation or reduction of molecular oxygen, 2-oxoglutarate as one donor, and incorporation of one atom each of oxygen into both donors” (GO:0016706), “naringenin 3-dioxygenase activity” (GO:0045486) and “leucocyanidin oxygenase activity” (GO:0050589). Catalytic activity also included transferase activity which, in turn, included “glucosyltransferase activity” (GO:0046527), “anthocyanidin 3-O-glucosyltransferase activity” (GO:0047213) and “transferase activity, transferring acyl groups other than amino-acyl groups” (GO:0016747) and “hydroxycinnamoyltransferase activity” (GO:0050734). Binding included “heme binding” (GO:0020037), “cofactor binding” (GO:0048037), including “FAD binding” (GO:0050660) as well as “transcription factor activity” (GO:0003700).

It is interesting to note that the enriched term “transcription factor activity” included several ethylene-responsive factors, such as a homologue of tomato ethylene responsive transcriptional coactivator (MBF1), AP2/ERF domain-containing contigs, homologues of dehydration-responsive element binding protein, and ethylene-responsive transcription factor 2 (ERF2) and ERF5, indicating involvement of ethylene in sweetpotato root development. The growth of secondary xylem and phloem depends on the division of cells in the vascular cambium and results in an increase in the diameter of the root. Results of genome-wide expression profiling of xylem and phloem cambium isolated from the root hypocotyl of *Arabidopsis* suggested a role for several members of the AP2 as well as MYB transcription factor families, in addition to other transcription factors, as regulators of xylem or phloem cell differentiation and activity [47]. This “transcription factor activity” GO term also contained R2R3 MYB class transcription factor homologues, several GRAS2 and GRAS10 homologues, two BEL1 homeotic protein homologues and a CONSTANS-like protein homologue. Members of the GRAS family, such as the short-root protein, are key regulators of root radial patterning [[20] and references therein], meristem maintenance and asymmetric cell division [[20] and references therein], and have been found to be differentially expressed in SRs of the sweetpotato cv. *Xushu*[20].

The GO terms “large ribosomal subunit” (GO:0015934), “extracellular region” (GO:0005576) and “intrinsic to membrane” (GO:0031224) were found to be significantly enriched in the CC category (Figure 10C).

#### ***Analysis of KEGG pathway enrichment in differentially expressed contigs of either ISRs or FRs, relative to the root transcriptome database***

The differentially expressed contigs in ISRs and FRs were analyzed for KEGG pathway enrichment, relative to the rest of the root transcriptome database, using Fisher’s Exact Test and FDR correction [48], and the results are presented in Table 4. Four and fifteen pathways were found to be significantly enriched (FDR ≤ 0.05) in ISR and FR, respectively.

**Table 4:** KEGG pathway enrichment in initiating storage roots and fibrous roots, relative to the rest of the root transcriptome database

**Sample**	**Pathway term**	**FDR**	**KEGG map**
**Fibrous roots**	Flavonoid biosynthesis	2.3E-17	map00941
	Phenylpropanoid biosynthesis	5.3E-15	map00940
	Anthocyanin biosynthesis	1.2E-11	map00942
	Stilbenoid, diarylheptanoid and gingerol biosynthesis	3.8E-10	map00945
	Drug metabolism - other enzymes	1.1E-05	map00983
	Isoquinoline alkaloid biosynthesis	1.7E-04	map00950
	Linoleic acid metabolism	6.7E-04	map00591
	Phenylalanine metabolism	5.3E-03	map00360
	alpha-Linolenic acid metabolism	7.0E-03	map00592
	Zeatin biosynthesis	1.1E-02	map00908
	Cysteine and methionine metabolism	1.2E-02	map00270
	Flavone and flavonol biosynthesis	2.4E-02	map00944
	Propanoate metabolism	2.5E-02	map00640
	Ubiquinone and other terpenoid quinone biosynthesis	4.4E-02	map00130
	Betalain biosynthesis	5.2E-02	map00965
**Initiating storage roots**	Galactose metabolism	0.000	map00052
	Sulfur metabolism	0.001	map00920
	Fructose and mannose metabolism	0.001	map00051
	Glycolysis/Gluconeogenesis	0.051	map00010

Enrichment analysis was done using Fisher’s Exact Tests. P-value is given as an adjusted value following FDR correction (FDR ≤ 0.05).

Looking into KEGG-enriched pathways in ISRs, enrichment of sulfur metabolism was apparent, with 10 enzymes exhibiting up-regulated expression in ISRs compared to FRs (Additional file 11), including contigs representing sulfite reductase (ferredoxin). Interestingly, sulfite reductase has been recently suggested as a candidate gene with potential function in cassava SR formation [49]. Reduced levels of sulfite reductase in the *Arabidopsis* mutant *sir1* with decreased activity of the enzyme led to decreased hexose and starch contents [50]. It is thus tempting to suggest that sulfite reductase may also have a role in the control of SR starch accumulation during SR initiation. The glycolysis/gluconeogenesis pathway was also found to be significantly enriched in ISRs (FDR = 0.051; Table 4). In cassava, regulatory changes in the glycolysis/gluconeogenesis pathway were demonstrated following SR development [51]. Down-regulation of enolase, L-lactate dehydrogenase and aldehyde dehydrogenase was suggested in cassava to slow down the entry of carbon into the citrate cycle, pyruvate metabolism and propanoate metabolism, leading to less glucose-6P converted to glycerate-3P and glucose-1P, and in most of the glucose-6P being transported into the amyloplast for starch and sucrose synthesis [51]. In sweetpotato, over twofold down-regulation of several contigs of pyruvate decarboxylase and lactate dehydrogenase was detected following initiation of SR formation (Additional file 5), as well as more than twofold up-regulation of contigs of phosphoglucomutase (catalyzing the reversible interconversion between glucose-6P and glucose-1P, the latter serving as a substrate for ADP-Glc pyrophosphorylase, the first committed step in the starch biosynthesis pathway), indicating the possibility of similar regulation.

**Figure 11:**
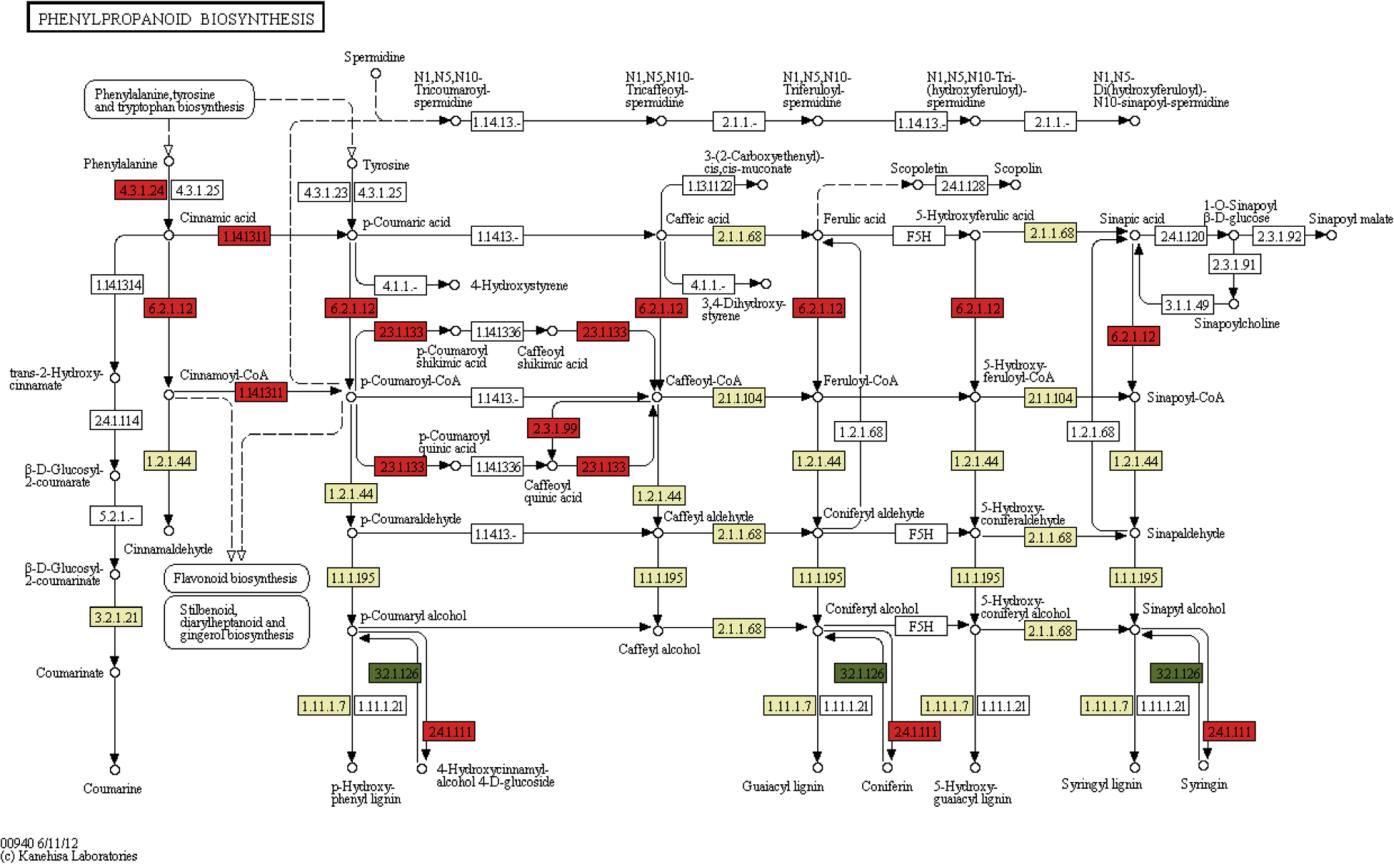
**Changes in the phenylpropanoid biosynthesis pathway map between fibrous roots (FRs) and initiating storage roots (ISRs).** Enzymes exhibiting up-regulated expression in ISRs and FRs are marked in green and in red, respectively. Marked in light green are enzymes representing gene sequences that exhibit up-regulated expression in both ISRs and FRs (in most cases, a larger number of contigs exhibited higher expression in FRs compared to ISRs (Table 5)). Marked in white are enzymes representing gene sequences that were not detected in the Illumina-generated transcription profiles (exhibited less than 10 reads) or general enzyme categories representing an enzyme class (such as 4.1.1.-, 2.1.1- and 5.2.1-, representing lyases, methyltransferases and isomerases, respectively). Enzyme annotation was obtained from the sequence annotation and GO classification data.

Among the significantly enriched pathways in FRs, high enrichment was observed for the phenylpropanoid biosynthesis pathway (Table 4). The first three biosynthetic reactions in this pathway, referred to as the general phenylpropanoid pathway, produce p-coumaroyl CoA, which is a major branch-point metabolite between the production of the flavonoids and the pathway that produces monolignols, lignans and hydroxy-cinnamate conjugates [[52] and references therein]. The first of these reactions is the deamination of phenylalanine by phenylalanine ammonia-lyase (PAL) to generate trans-cinnamic acid. Cinnamic acid is then para-hydroxylated by cinnamate 4-hydroxylase (C4H) to produce p-coumaric acid [[52] and references therein], which is then activated to its corresponding CoA thioester by 4-coumarate CoA ligase (4 coumaroyl-CoA synthase; 4CL). All phenylalanine-derived units destined to be incorporated into the lignin polymer must be hydroxylated by C4H, because the p-hydroxy group is required for the activation of monolignols to their corresponding free radicals, and for polymerization into lignin. The phenylpropanoid biosynthesis pathway map is presented in Figure 11, with the enzymes exhibiting up-regulated expression in ISRs and FRs marked in green and red, respectively. Over twofold up-regulation in FRs of contigs representing C4H and 4CL, as well as of contigs of coniferyl-alcohol glucosyltransferase was apparent (Figure 11 and Table 5). In addition, high expression of PAL was detected in the FR sample, whereas an over fourfold reduction in read number was observed in the ISR sample (Additional file 5). The presence of several contigs representing these enzymes may indicate the presence of isoenzymes. 4CL catalyzes the formation of CoA esters of caffeic acid, ferulic acid, 5-hydroxyferulic acid, and sinapic acid, in addition to p-coumaric acid [[53] and references therein]. The plethora of additional potential substrates may explain why there are many 4CL isoenzymes in most plants. In addition to the different substrate specificities, the genes may have a distinct spatiotemporal expression pattern [[53] and references therein]. Looking into the read number of contigs representing genes of the lignin pathway, such as cinnamyl alcohol dehydrogenase (CAD), more than fivefold lower expression was detected in the ISR vs. FR sample (Table 5 and Additional file 5). Taken together, the results indicate down-regulation in the expression of key genes of the phenylpropanoid biosynthesis pathway upon the change in root fate from FR to a storage organ, which may be responsible for the significant reduction in lignin levels (Figure 1), representing novel data not previously described in sweetpotato. Indeed, it has been demonstrated in *Arabidopsis* and tobacco that down-regulating 4CL results in reduced lignin content [54-56]. Hu et al. [57] showed that down-regulating the expression of 4CL in transgenic aspen (*Populus tremuloides* Michx.) by antisense inhibition causes up to 45% reduction in lignin. Reductions in lignin content in *Arabidopsis* plants carrying a mutation in the second enzyme of this pathway, C4H, were shown to accumulate decreased levels of several different classes of phenylpropanoid end products and to exhibit reduced lignin deposition, altered lignin monomer content and a collapsed xylem phenotype [52].

**Table 5:** Read count of contigs representing enzymes of the phenylpropanoid biosynthesis pathway in initiating storage roots and fibrous roots

**Enzyme/contigs**	**ISR reads**	**FR reads**
**phenylalanine ammonia-lyase (PAL; EC 4.3.1.24)**		
S_PBL_c14873	230.1	3407.1
S_PBL_c18	905.5	11467.6
S_PBL_c18502	387.3	2829.8
S_PBL_2066	521.0	7128.4
S_PBL_21573	17.8	54.5
S_PBL_2312	344.2	3051.1
**trans-cinnamate 4-monooxygenase (C4H; EC 1.14.13.11)**		
S_PBL_lrc53776	125.3	2954.8
S_PBL_lrc53138	10.3	116.5
S_PBL_lrc53079	262.9	1090.4
S_PBL_lrc52110	345.2	6252.8
S_PBL_c98	434.0	4534.9
S_PBL_c2889	391.0	9808.5
S_PBL_c22453	285.3	1095.8
S_PBL_c18141	154.3	2287.8
S_PBL_c17334	707.2	2703.6
**4-coumarate-CoA ligase (4CL; EC 6.2.1.12)**		
S_PBL_c4746	63.6	421.2
S_PBL_c24867	50.5	162.5
S_PBL_c22722	70.2	388.1
S_PBL_c21439	56.1	305.7
S_PBL_c18044	29.9	379.5
S_PBL_c158	119.7	1904.0
S_PBL_c1395	52.4	149.7
S_PBL_c13368	18.7	56.7
**quinate O-hydroxycinnamoyltransferase (EC 2.3.1.99)**		
S_PBL_c20232	8.4	90.9
**shikimate O-hydroxycinnamoyltransferase (HCT; EC 2.3.1.133)**		
S_PBL_c8321	185.2	789.0
S_PBL_c657	3.7	93.0
S_PBL_c6051	58.0	346.4
S_PBL_c20232	8.4	90.9
S_PBL_c20009	85.1	1152.4
S_PBL_c17752	33.7	318.6
S_PBL_c15462	13.1	43.8
**caffeoyl-CoA O-methyltransferase (CCoAOMT; EC 2.1.1.104)**		
S_PBL_c6206	218.0	645.7
S_PBL_c3822	73.9	811.4
S_PBL_c2944	389.1	1204.8
S_PBL_c20480	148.7	1188.8
S_PBL_c12810	89.8	916.2
S_PBL_c30794	50.5	4.3
**cinnamoyl-CoA reductase (CCR; EC 1.2.1.44)**		
S_PBL_c8128	116.9	330.3
S_PBL_c5810	10499.1	30368.4
S_PBL_c2607	219.8	988.9
S_PBL_c21123	86.1	495.0
S_PBL_c19132	27.1	164.6
S_PBL_c10515	42.1	10.7
**caffeate O-methyltransferase (COMT; EC 2.1.1.68)**		
S_PBL_c978	98.2	405.2
S_PBL_c538	26.2	327.1
S_PBL_c20536	2.8	20.3
S_PBL_c29777	78.6	15.0
**cinnamyl-alcohol dehydrogenase (CAD; EC 1.1.1.195)**		
S_PBL_lrc54926	37.4	95.1
S_PBL_lrc54183	23.4	136.8
S_PBL_lrc53688	632.3	8881.6
S_PBL_lrc53173	17.8	96.2
S_PBL_lrc52706	599.6	2211.9
S_PBL_lrc52587	355.5	2451.3
S_PBL_lrc26619	6.5	21.4
S_PBL_c8094	178.7	1763.9
S_PBL_c27372	114.1	351.7
S_PBL_c22346	11.2	49.2
S_PBL_c21896	27.1	161.4
S_PBL_c21084	474.3	4448.3
S_PBL_c18369	225.4	776.1
S_PBL_c17373	29.0	139.0
S_PBL_c12615	328.3	2762.4
S_PBL_c20863	186.1	18.2
S_PBL_c17269	509.8	160.4
**beta-glucosidase (EC 3.2.1.21)**		
S_PBL_c6163	4.7	13.9
S_PBL_c41088	0.9	35.3
S_PBL_c29128	1.9	63.1
S_PBL_c25813	39.3	101.6
S_PBL_c12164	62.7	374.2
S_PBL_c13042	12.2	3.2
S_PBL_c23506	64.5	2.1
S_PBL_c7311	353.6	127.2
S_PBL_c25672	55.2	8.6
S_PBL_c11889	156.2	44.9
S_PBL_c50698	50.5	4.3
S_PBL_c27348	74.8	23.5
**peroxidase (EC 1.11.1.7)**		
S_PBL_lrc55044	107.6	314.3
S_PBL_lrc54488	8.4	81.2
S_PBL_lrc54403	413.5	1216.6
S_PBL_lrc54045	769.8	1999.1
S_PBL_lrc53204	427.5	1603.6
S_PBL_lrc53085	334.9	1066.9
S_PBL_lrc52151	4.7	24.6
S_PBL_c8312	44.9	1339.5
S_PBL_c750	82.3	267.3
S_PBL_c6142	1.9	275.8
S_PBL_c5935	2.8	23.5
S_PBL_c5844	586.5	2139.2
S_PBL_c4994	61.7	609.4
S_PBL_c48762	40.2	985.7
S_PBL_c44935	2.8	13.9
S_PBL_c39736	11.2	40.6
S_PBL_c34320	2.8	65.2
S_PBL_c29852	223.6	602.9
S_PBL_c29801	24.3	215.9
S_PBL_c29652	0.9	151.8
S_PBL_c2946	92.6	280.1
S_PBL_c22233	5.6	22.4
S_PBL_c22191	5.6	20.3
S_PBL_c2122	629.5	1831.3
S_PBL_c21120	1.9	88.7
S_PBL_c18854	25.3	93.0
S_PBL_c1825	22.4	182.8
S_PBL_c16766	14.0	60.9
S_PBL_c1640	39.3	2253.6
S_PBL_c15871	156.2	1221.9
S_PBL_c12624	42.1	143.3
S_PBL_c11598	79.5	299.3
S_PBL_c11410	97.3	457.6
S_PBL_c14485	116.9	32.1
S_PBL_c15642	66.4	9.6
S_PBL_c7505	95.4	32.1
S_PBL_c2035	1005.6	360.3
S_PBL_c11972	18.7	4.3
S_PBL_lrc54799	75.8	29.9
S_PBL_c4878	34.6	1.1
S_PBL_c4857	549.1	97.3
S_PBL_c10185	100.1	31.0
S_PBL_c17430	18.7	3.2
S_PBL_lrc54785	52.4	8.6
**coniferyl-alcohol glucosyltransferase (EC 2.4.1.111)**		
S_PBL_c1688	61.7	420.1
S_PBL_c16389	61.7	1024.2
S_PBL_c13307	580.9	4191.7
**coniferin beta-glucosidase (EC 3.2.1.126)**		
S_PBL_c25672	55.2	8.6

*ISR*, Initiating storage root; *FR*, Fibrous root. Enzyme annotation was obtained from the sequence annotation and GO classification data.

Cross-talk between primary and secondary metabolism is well documented [58]. To activate the lignin biosynthesis pathway, carbon flow should be delivered from carbohydrate metabolism into the phenylpropanoid pathway by producing sufficient phenylalanine via the shikimate pathway. 3-Deoxy-D-arabino-heptulosonate-7-phosphate synthase (EC 2.5.1.54) catalyzes the first step of the shikimate pathway, using phosphoenolpyruvate and erythrose 4-phosphate derived from glycolysis and the pentose phosphate pathway, respectively. It was thus interesting to note that more than fourfold down-regulation in read number of six contigs (S_PBL_c17757, S_PBL_lrc54453, S_PBL_lrc54372, S_PBL_lrc55028, S_PBL_c7135, S_PBL_c8050) representing this gene, was detected in ISR compared to FR (Additional file 5), suggesting reduction in carbon flow toward phenylpropanoid biosynthesis upon SR initiation.

## Conclusions

In the present study, the generation of a database of *Georgia Jet* sweetpotato root transcriptome, together with a comparison of the expression profiles of ISRs and FRs, enabled the identification of genes involved in the earliest stage of SR formation. Down-regulation in the expression of key genes of the phenylpropanoid biosynthesis pathway upon the change in root fate from FR to a storage organ is indicated, which may be responsible for the significant reduction in lignin levels, representing novel data not previously described in sweetpotato.

The results highlight a reduction in carbon flow toward phenylpropanoid biosynthesis and its delivery into carbohydrate metabolism and starch biosynthesis as major events involved in SR initiation. Specific genes in these pathways were pointed out, providing potential targets for sweetpotato genetic engineering. The results also emphasize the potential importance of delicate control at the level of gene expression of regulators of meristematic tissue identity and maintenance, up-regulation of cell-division regulators and down-regulation of specific GRAS family members, in the SR-initiation process, providing novel data with respect to the specific genes involved. In addition, this study adds a valuable resource of sweetpotato root transcript sequences to the available data, facilitating the identification of genes of interest in this food crop which is among the top seven most important food crops in the world.

## Methods

### Plant material and RNA extraction

Georgia Jet is the most important sweetpotato variety grown in Israel. Fifty Georgia Jet plants were produced from transplants and grown in 30-cm pots filled with unfertilized washed sand in a greenhouse at The Volcani Center, Israel, maintained between 22 and 28°C with no supplemental light. Roots from 20 plants were sampled 3 to 4 weeks after transplanting as detailed in Villordon et al. [7] to identify the timing of SR initiation (marked by initiation of AC cell development). In short, adventitious roots were sectioned at the proximal 3-cm section of the root and the transverse sections were stained with toluidine blue and observed under the microscope. SR initiation was recorded at 4 weeks after transplanting. For each of the remaining 30 plants at this time point, all adventitious roots were sectioned for microscopic analysis and the adjacent root tissue was immediately frozen by plunging into liquid nitrogen. Following microscopic analysis, roots were divided into either ISRs or non-initiated FRs, as shown in Figure 1, and pooled into ISR and FR samples. Root tissue was ground to a fine powder using liquid nitrogen and sea sand (Merck, Darmstadt, Germany), and total RNA was extracted using the Tri reagent (Sigma-Aldrich, Rehovot, Israel). RNA was treated with TURBO DNase (AB Applied Biosystems, Ambion, CA, USA) according to the manufacturer’s instructions. The two total RNA samples were examined by capillary electrophoresis using a Shimadzu MultiNA microchip electrophoresis system, and used for the preparation of two types of cDNA libraries as detailed below.

### Preparation of a normalized random-primed cDNA library for 454 sequencing

For cDNA synthesis, the two RNA samples were pooled in equal amounts to form a pool designated ISR_FR. A normalized cDNA library was constructed (Eurofins MWG operon, Ebersberg, Germany: http://www.eurofinsgenomics.eu/). In brief, from the DNase-treated RNA pool, poly(A) + RNA was isolated and used for cDNA synthesis. First-strand cDNA synthesis was primed with a N6 randomized primer. Then 454 adapters A and B were ligated to the 5′ and 3′ ends of the cDNA. The cDNA was amplified with 11 PCR cycles using a proofreading enzyme (Additional file 12A, N0). Normalization was carried out by one cycle of denaturation and reassociation of the cDNAs, resulting in N1-cDNAs. Reassociated ds-cDNA was separated from the remaining ss-cDNA (normalized cDNA) by passing the mixture over a hydroxylapatite column. The ss-cDNAs were then amplified with nine PCR cycles (Additional file 12A, N1). For titanium sequencing, the 600- to 800-bp cDNAs were eluted from a preparative agarose gel. Aliquots of the size-fractionated cDNAs were analyzed by capillary electrophoresis (Additional file 12B). The 600- to 800-bp ds-cDNA exhibited the structure described in Additional file 13.

### Sequencing by Roche GS FLX technology, using titanium series chemistry

Following elution from the preparative gel, this size-selected cDNA was sequenced using a Genome Sequencer™ (GS) FLX Titanium Instrument (Roche Diagnostics) following a standard protocol [59]. The 454 Life Sciences (Roche Diagnostic) software was used for image and signal processing. A file containing the trace, “base-calling” and quality score data was generated and stored in standard flowgram format (SFF) for subsequent bioinformatic analyses. The sequence data was deposited into the European Nucleotide Archive [EMBL-EBI: PRJEB4145; sample accession ERS255740]. An automated, in-silico-assembly pipeline (Eurofins MWG Operon) was used to assemble the sequence data de novo. FASTA and associated files of 524,607 high-quality, base-called and clipped reads were extracted from the SFF-file and contigs assembled de novo using MIRA (version 2.9.45 × 1; http://chevreux.org/projects_mira.html; [60]). Mean lengths ± SD in bases were calculated for particular nucleotide sequence data subsets. MIRA assembly clustered the reads into 55,296 contigs.

### Preparation of non-normalized 3’-fragment cDNA libraries from each of the two root samples and Illumina GA-II sequencing

The extracted total RNA from each of the two pooled root samples—ISRs and non-initiated FRs—was used for cDNA synthesis. The cDNA libraries were prepared by Eurofins MWG Operon. In brief, total RNAs were first sheared with ultrasound (10 pulses of 30 s at 4°C), then poly(A) + RNA was isolated followed by treatment of the RNA fragments with polynucleotide kinase. An RNA adapter was then ligated to the 5′-phosphate of the 3’-terminal RNA fragments. First-strand cDNA synthesis was performed using an oligo(dT)-adapter primer and MMLVH- reverse transcriptase. The resulting cDNAs were PCR-amplified (number of cycles indicated in Additional file 14A) using high-fidelity DNA polymerase. Barcode sequences, which were attached to the 5′-ends of the cDNAs, are described in Additional file 14A. The cDNAs in the size range of 200–450 bp were eluted from preparative agarose gels. Aliquots of the size-fractionated cDNAs were analyzed by capillary electrophoresis (Additional file 14B). The resulting, ds-cDNAs were of about 200–450 bp as demonstrated in Additional file 14C and contained adapter sequences (Additional file 14D). Illumina sequencing using the GA-II platform was performed by Eurofins MWG operon according to the manufacturer’s instructions (Illumina, San Diego, CA, USA). A quality score of Q30 was used [Q30 being associated with an error rate (namely the probability that a base is called erroneously) of 0.1%]. The specifications concerning Q30 were as follows: ≥70% of the reads having Q30 for reads longer than 75 bases and ≥75% of the reads having Q30 for reads up to 50 bases.

### Illumina read mapping against the 454-generated sweetpotato root transcript sequences

The raw Illumina reads were sorted using their tags (ISR: GAGT and FR: CTTG; Additional file 14A), resulting in the following number of reads per sample (HiSeq 2000, 1 × 100 bp): 14,780,229 and 17,703,982 for ISR and FR, respectively. The sequence data was deposited into the European Nucleotide Archive [EMBL-EBI: PRJEB4145; sample accession numbers ERS255744 and ERS255745 for ISRs and FRs, respectively]. Prior to the read mapping, the four tag bases were removed from its 5′ end. These read sequences were then used as input for read mapping against the set of 55,296 FLX EST contigs. The mapping was conducted using the software BWA 0.5.8c (http://bio-bwa.sourceforge.net). Post-processing of the mapping was conducted with samtools 0.1.12a (http://samtools.sourceforge.net). The total number of mapped reads for each of the two samples (ISR and FR) was quantified. To enable a direct comparison between the two samples, the read count per EST contig was normalized using DESeq [61].

### Validation of the Illumina-generated transcription profiles of FR and ISR samples using real-time qRT-PCR analyses

RNA samples were prepared from either FRs or ISRs as described above, using at least four biological replicates. Each replicate contained root tissue derived from 30 plants. RNA samples were cleaned of DNA contamination using RQ1 RNase-free DNase (Promega WI, USA). cDNA was synthesized using 1 μg of total RNA and Superscript II (Invitrogen) reverse transcriptase, according to the manufacturer’s instructions. First-strand cDNA was used for qRT-PCR analyses. Primer3web version 4.0: http://primer3.ut.ee) was used for primer design, and qRT-PCR was performed on the ROTOR-GENE™ 6000 (Corbett LIFE SCIENCE, Qiagen), using SYBR^®^ Green. A total reaction volume of 15 μl was used. The reaction mix included 3 μl template, 0.3 μl reverse primer, 0.3 μl forward primer, 7.5 μl Absolute Blue QPCR SYBR Green ROX Mix (Thermo Fisher Scientific Inc. MA, USA), and 3.9 μl RNA-free water. A qRT-PCR assay was performed using the following conditions: 95°C for 9 min followed by 40 cycles of 95°C for 10 s, 60°C for 10 s and 72°C for 20 s. The 2^–ΔΔCT^ method [62] was used to normalize and calibrate transcript values relative to the 18S ribosomal protein, whose expression did not change across sweetpotato root types or developmental stages. The FR sample was used as the calibrator sample. Primer sequences were designed according to the respective contigs assembled from reads that were obtained from the 454-sequencing results and are described in Additional file 15. Use of the oligonucleotide primers listed in Additional file 15 resulted in approximately equal efficiencies of amplification for the various target and reference genes. Each set of experiments was repeated at least four times and final relative quantification results are given as average ± SE.

### Functional annotation and analyses of GO-term and KEGG-pathway enrichment

The assembled transcripts were used to query public genomic databases (i.e. NCBI Nr) using BLASTX (Eurofins MWG Operon) and annotations of the two best hits for each contig were recorded. BlastoGO [22] was used to obtain GO annotations and Ontologizer [23] was used to perform GO functional classification. The contigs were further classified using GOSlim (http://www.geneontology.org/GO.slims.shtml). To assign the detected contigs to biological pathways, sequences were compared using BLASTX with an E-value cutoff of <10E-3 against the KEGG database (http://www.genome.jp/kegg/). The differentially expressed contigs in ISRs and FRs were analyzed for GO-category enrichment relative to the root transcriptome database using AgriGO [44]. The differentially expressed contigs in ISRs and FRs were analyzed for KEGG-pathway enrichment relative to the rest of the root transcriptome database using Fisher’s Exact Test and FDR correction [48].

### Starch content analysis

Root samples of *Georgia Jet* were pooled from five to seven plants at 1, 2, 3 and 4 weeks after transplanting, spanning the period of SR initiation [7]. The tissue was ground in liquid nitrogen using a mortar and pestle and ethanol-suspended root samples were extracted three times in hot 80% (v/v) ethanol. The insoluble residue that remained after ethanolic extraction was resuspended in 30 mM HCl and boiled for 30 min. After cooling, the pH was adjusted to 4.5 with KOH. The gelatinized starch was digested for 60 min at 50°C with approximately 36 U of amyloglucosidase from *Aspergillus oryza*[63]. Reducing sugars were determined according to Miller [64]. Three biological replicates were used for each analysis.

## Abbreviations

AC: Anomalous cambium; DAT: Days after transplanting; FR: Fibrous root; ISR: Initiating storage root; LC: Lignified cell; NGS: Next-generation sequencing; RVC: Regular vascular cambium; SR: Storage root.

## Competing interests

The authors declare that they have no competing interests.

## Authors’ contributions

The study was conceived by NF, DL and AV. NF drafted the manuscript. YK designed the experimental setup, together with NF, DL and AV, and performed the experiments, including tissue sections, RNA extractions and starch content analyses. EL performed the quantitative RT-PCR analyses. JS contributed to the experiment performance and data analysis. TSP performed the bioinformatics analyses, including functional annotation of the database. ADF participated in the bioinformatics analyses, including GO category and KEGG pathway enrichment. AH generated the sweetpotato transcriptome web page. L Althan contributed to the plant material preparation and maintenance. L Adani Nadir participated in RNA extractions. All authors read and approved the final manuscript.

## Supplementary Material

Additional file 1 **Annotations of the two best hits for each contig (E value ≤ 1.0E-3).** Annotations are listed in boxes I2 – I61,668. Click here for file

Additional file 2 **Species distribution of the BLAST results.** Presented data were extracted from the detailed data of the number of BLAST results matching each plant species. For each contig, the best BLAST result was used. Click here for file

Additional file 3 **Functional annotations of sweetpotato root transcripts (combined initiating storage and fibrous root samples) using GOSlim.** The three GO functional categories (BP, MF, CC) were segregated into separate worksheets. Whenever relevant, contigs were assigned to more than one GO term. Click here for file

Additional file 4 Functional classification of sweetpotato root transcripts (combined initiating storage and fibrous root samples) to biological pathways using KEGG. Click here for file

Additional file 5 **Total normalized read counts in the fibrous root and initiating storage root samples.** Illumina GA-IIx technology was carried out to compare the transcription profiles of a pooled sample of fibrous roots and a pooled sample of initiating storage roots and reads were mapped against our database of 55,296 FLX contigs. Normalization was performed as described in ‘Methods’. One annotation is given for each contig. Annotations of the two best hits for each contig (E value ≤ 1.0E-3) are available in Additional file 1. Click here for file

Additional file 6 **Differentially expressed contigs between initiating storage roots (ISRs) and fibrous roots (FRs).** A contig was considered as differentially expressed if two conditions were met: (1) at least 10 reads were mapped to this contig in at least one of the samples and (2) no reads were mapped to this contig in the other sample, or the fold-change between its read number in each sample was at least 2.5. The up-regulated contigs in ISR and FR are segregated into separate worksheets. Fold-change expression was calculated relative to the read number in the ISR sample. One annotation is given for each contig. Annotations of the two best hits for each contig (E value ≤ 1.0E-3) are available in Additional file 1. Click here for file

Additional file 7 **GO-term enrichment in the initiating storage root sample relative to the root transcriptome database.** The specific contigs for each GO term in categories biological process (BP), molecular function (MF) and cellular component (CC) are given on separate sheets. ISR – initiating storage root sample. Click here for file

Additional file 8 **Tree representation of enriched GO terms in the initiating storage root sample.** A more intense color indicates a higher number of contigs. ISR – initiating storage root sample. Click here for file

Additional file 9 **GO-term enrichment in the fibrous root sample relative to the root transcriptome database.**vThe specific contigs for each GO term in categories biological process (BP), molecular function (MF) and cellular component (CC) are given on separate sheets. FR – fibrous root sample. Click here for file

Additional file 10 **Tree representation of enriched GO terms in the fibrous root sample.** A more intense color indicates a higher number of contigs. FR – fibrous root sample. Click here for file

Additional file 11 Read number of contigs representing the initiating storage root-enriched sulfur metabolism in the KEGG pathway. Click here for file

Additional file 12 **Analysis of the PCR-amplified cDNAs on a Shimadzu MultiNA microchip electrophoresis system.** A. Analysis of the PCR-amplified N0 and N1 cDNAs. B. Analysis of the size-fractionated N1 cDNAs. M – 100 bp ladder. Click here for file

Additional file 13 **Description of the cDNA used for 454 sequencing.** The 454 adapter sequences are underlined. The first four bases of adapter primers A1 and B1 represent phosphorothioate-modified bases as specified by Roche. In addition, primer B1 is 5′-biotinylated. The barcode sequence is: CACACG. Click here for file

Additional file 14 **Properties of the non-normalized cDNA samples.** A. PCR amplification of the cDNA samples. B. Analysis of the PCR-amplified 3′-fragment cDNAs on the Shimadzu MultiNA microchip electrophoresis system. C. Analysis of the size-fractionated cDNAs on the Shimadzu MultiNA microchip electrophoresis system. D. Description of cDNA for Illumina sequencing. Illumina adapter sequences are underlined. M – 100 bp ladder; ISR – initiating storage root sample; FR – fibrous root sample. Click here for file

Additional file 15 List of oligonucleotide primer sequences used for the quantitative RT-PCR analyses. Click here for file
