# Supplementary figures and images for: Transcriptional profiling of sweetpotato (Ipomoea batatas) roots indicates down-regulation of lignin biosynthesis and up-regulation of starch biosynthesis at an early stage of storage root formation
Source: BMC Genomics. 2013 Jul 9;14:460. doi: 10.1186/1471-2164-14-460 (PMC3716973; doi:10.1186/1471-2164-14-460)

Top - hit species distribution

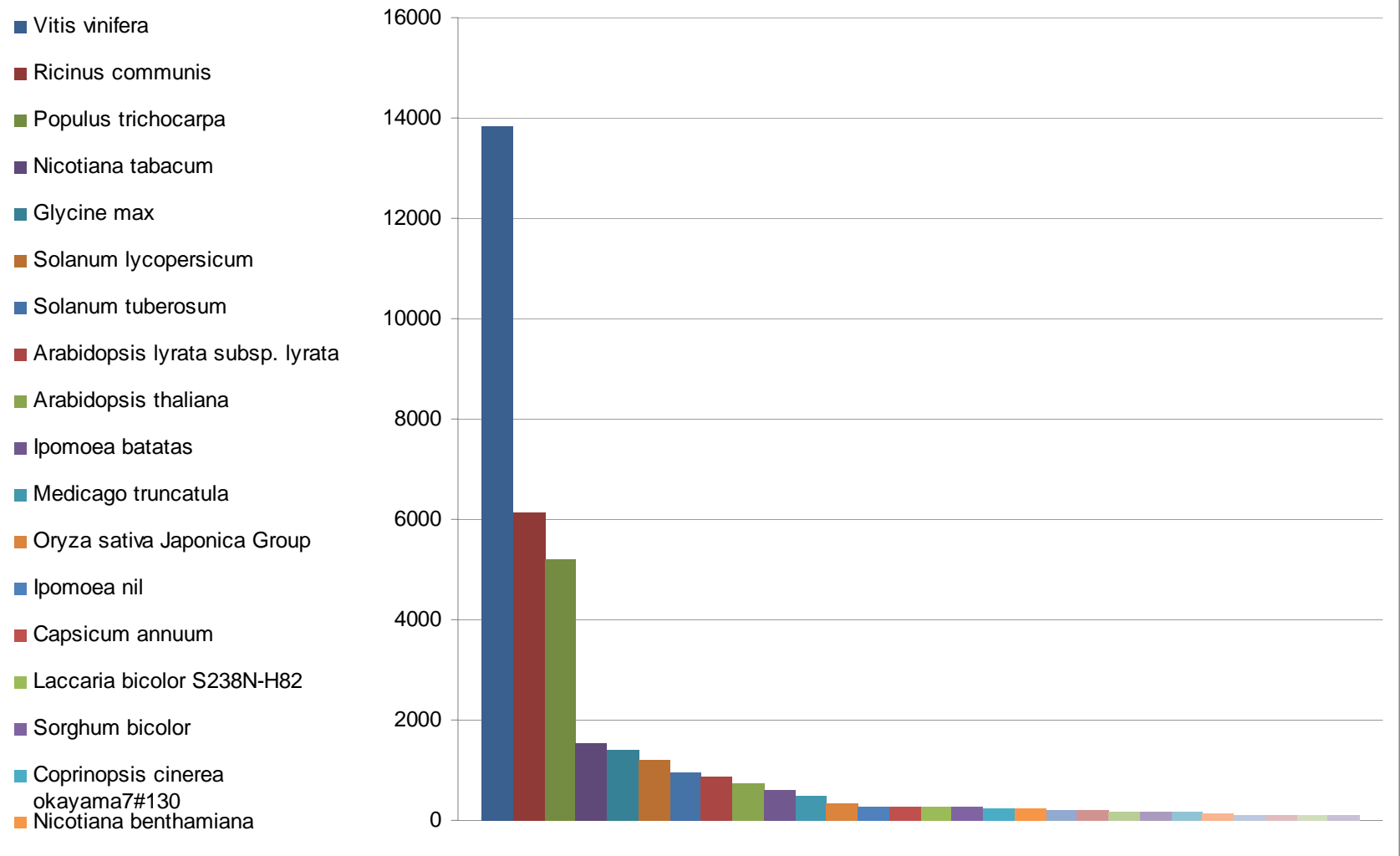

Supplement: Additional file 2 — Species distribution of the BLAST results. Presented data were extracted from the detailed data of the number of BLAST results matching each plant species. For each contig, the best BLAST result was used. [file 1471-2164-14-460-S2.pdf]

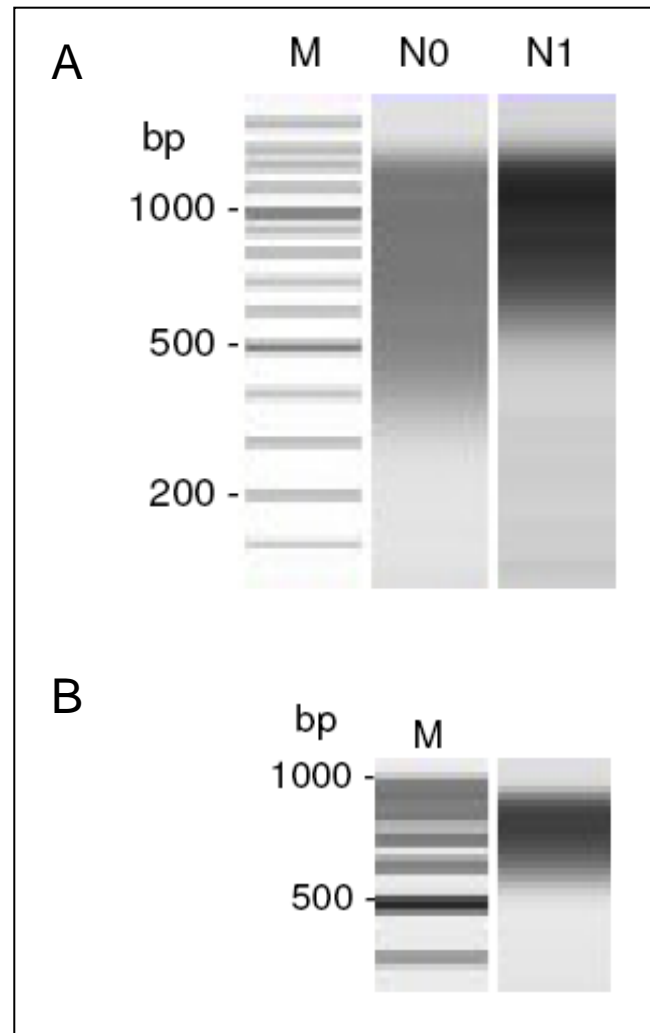

Supplement: Additional file 12 — Analysis of the PCR-amplified cDNAs on a Shimadzu MultiNA microchip electrophoresis system. A. Analysis of the PCR-amplified N0 and N1 cDNAs. B. Analysis of the size-fractionated N1 cDNAs. M – 100 bp ladder. [file 1471-2164-14-460-S12.pdf]
