# Supplementary material for: Transcriptional profiling of sweetpotato (Ipomoea batatas) roots indicates down-regulation of lignin biosynthesis and up-regulation of starch biosynthesis at an early stage of storage root formation
Source: BMC Genomics. 2013 Jul 9;14:460. doi: 10.1186/1471-2164-14-460 (PMC3716973; doi:10.1186/1471-2164-14-460)
Supplement: Additional file 8 — Tree representation of enriched GO terms in the initiating storage root sample. A more intense color indicates a higher number of contigs. ISR – initiating storage root sample. [file 1471-2164-14-460-S8.ppt]

## Slide 1
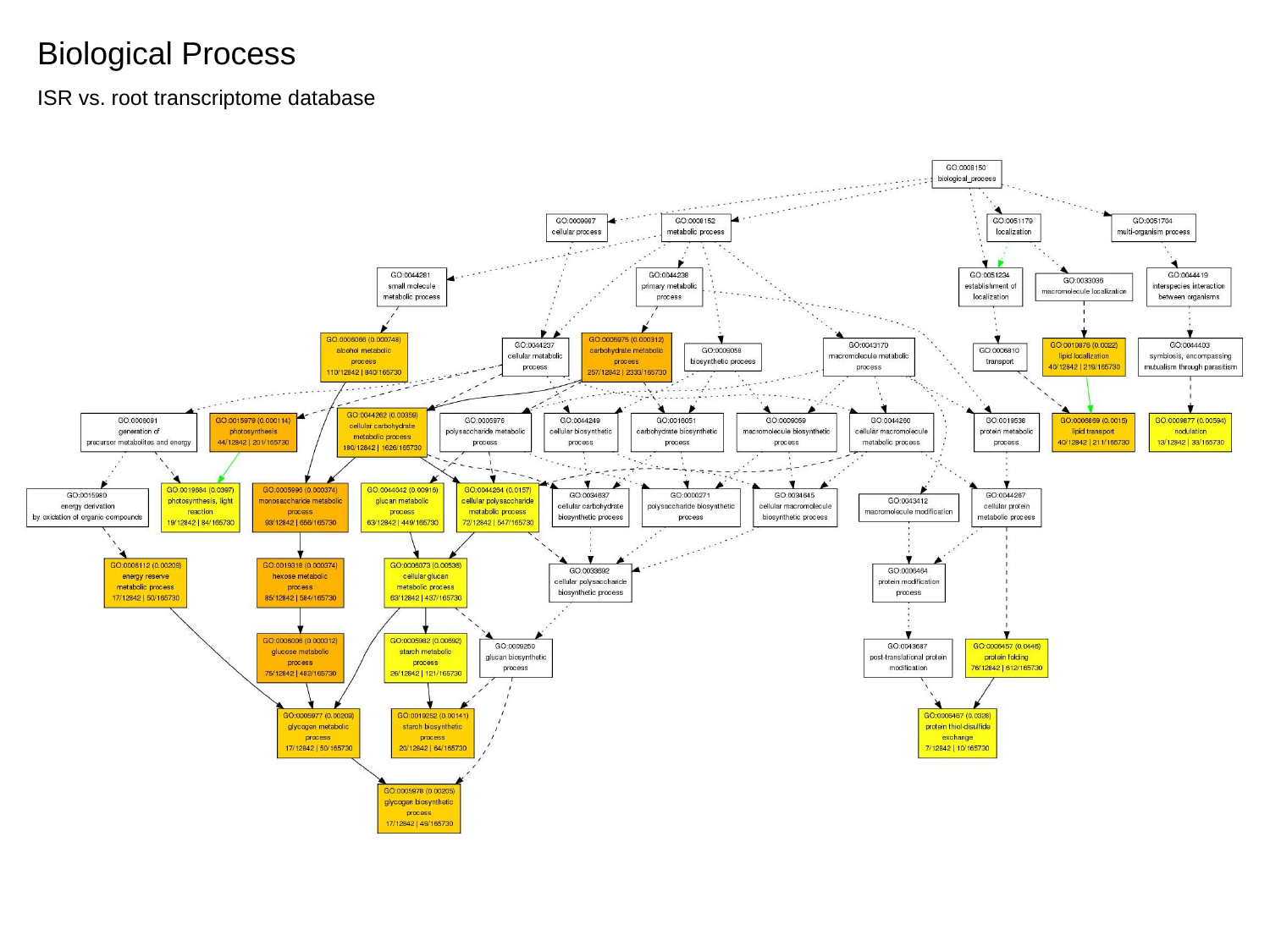

Biological Process
ISR vs. root transcriptome database

## Slide 2
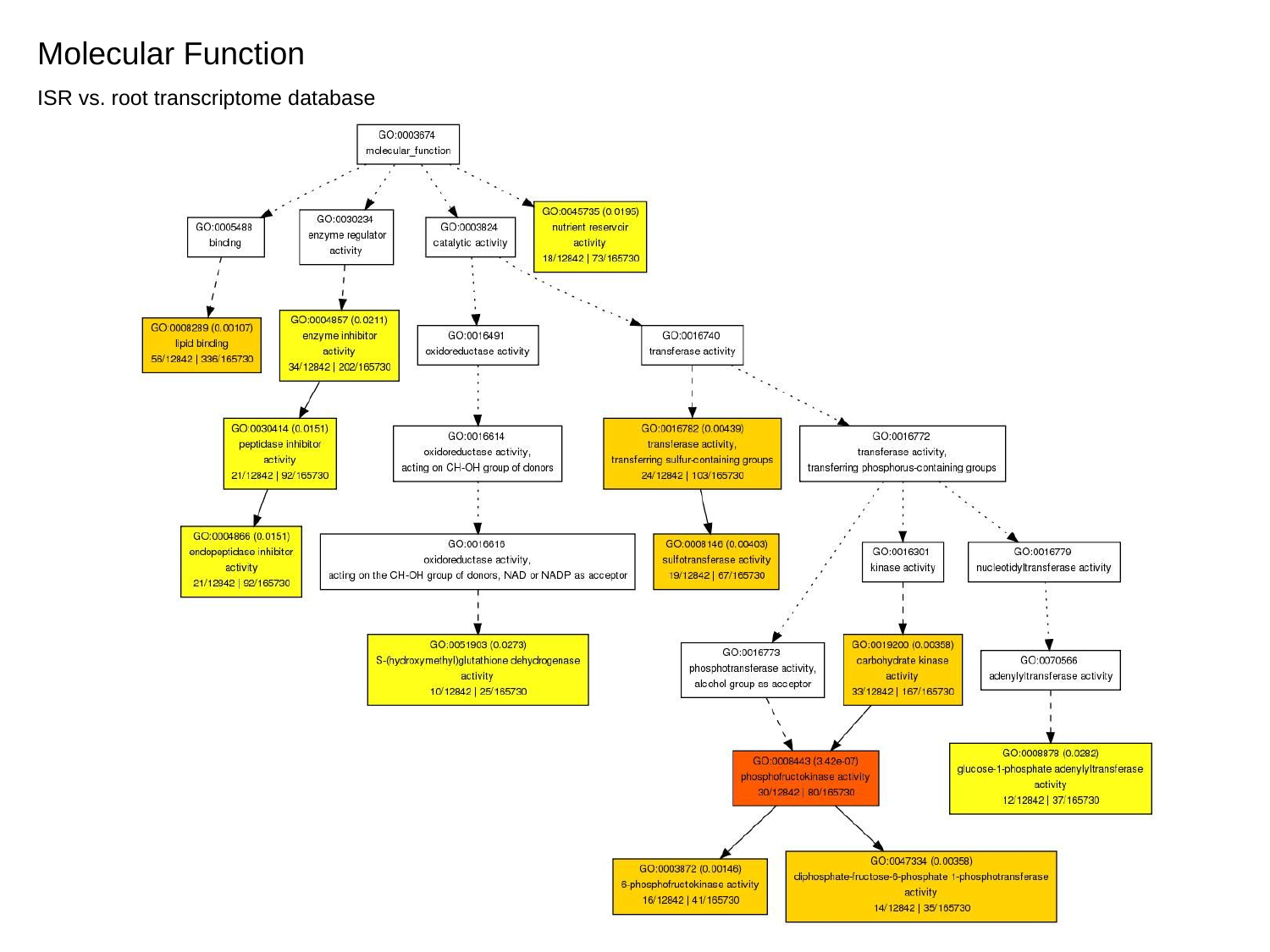

Molecular Function
ISR vs. root transcriptome database

## Slide 3
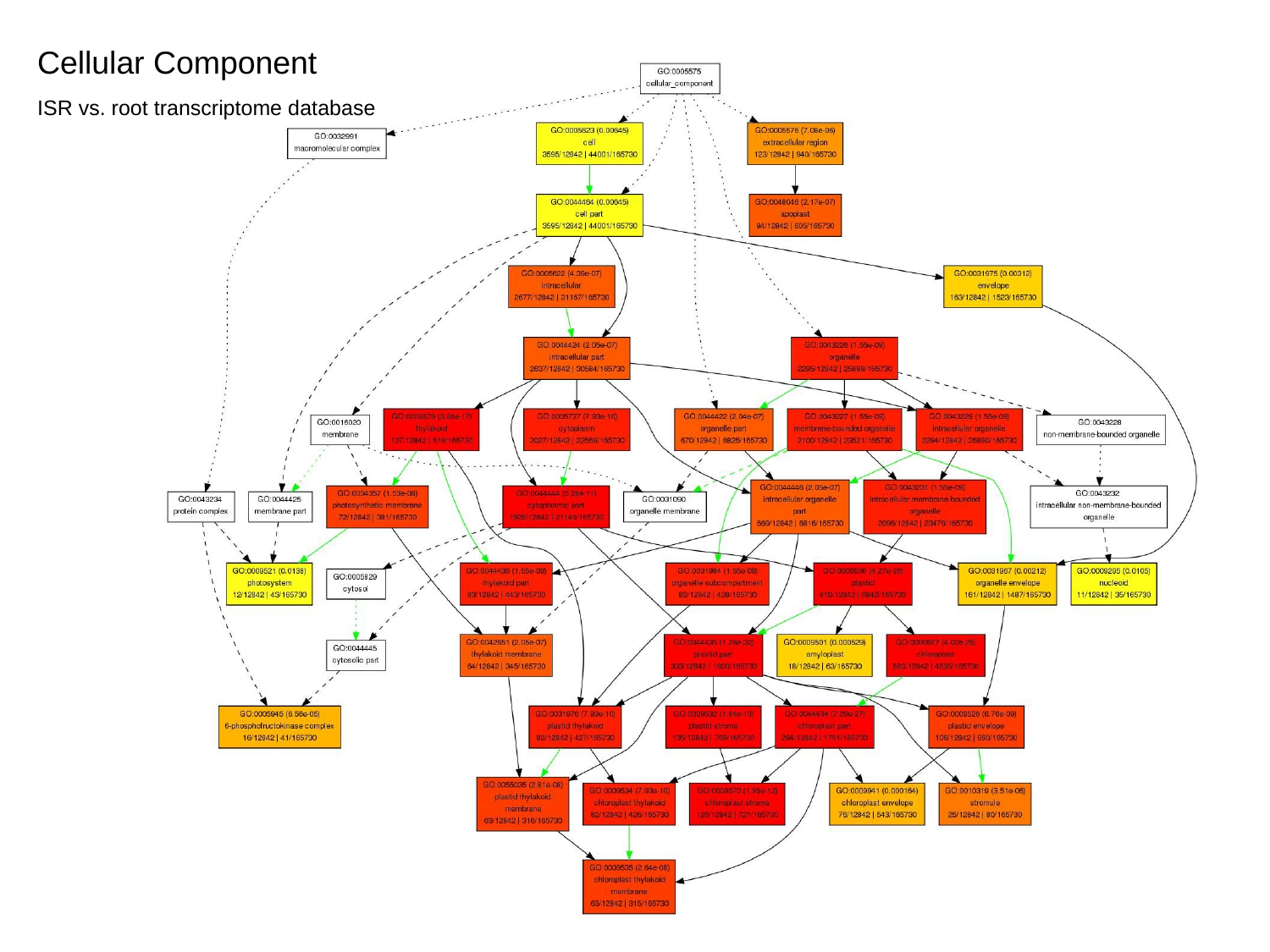

Cellular Component
ISR vs. root transcriptome database
