# Supplementary material for: Transcriptional profiling of sweetpotato (Ipomoea batatas) roots indicates down-regulation of lignin biosynthesis and up-regulation of starch biosynthesis at an early stage of storage root formation
Source: BMC Genomics. 2013 Jul 9;14:460. doi: 10.1186/1471-2164-14-460 (PMC3716973; doi:10.1186/1471-2164-14-460)
Supplement: Additional file 13 — Description of the cDNA used for 454 sequencing. The 454 adapter sequences are underlined. The first four bases of adapter primers A1 and B1 represent phosphorothioate-modified bases as specified by Roche. In addition, primer B1 is 5′-biotinylated. The barcode sequence is: CACACG. [file 1471-2164-14-460-S13.pdf]

5'-454 Adapter A1
CCATCTCATCCCTGCGTGTCTCCGACTCAG-

Barcode 5'-Adapter cDNA (500-700 bp) 3'-Adapter
NNNNNN-GACCTTGGCTGTCACTCAGTTNNNNNNNNNNNNNNNNNNNNNNNNNNNNNNTCGCAGTGAGTGACAGGCCA

3'-454 Adapter B1
- CTGAGACTGCCAAGGCACACAGGGGATAGG
