# Supplementary material for: Transcriptional profiling of sweetpotato (Ipomoea batatas) roots indicates down-regulation of lignin biosynthesis and up-regulation of starch biosynthesis at an early stage of storage root formation
Source: BMC Genomics. 2013 Jul 9;14:460. doi: 10.1186/1471-2164-14-460 (PMC3716973; doi:10.1186/1471-2164-14-460)
Supplement: Additional file 14 — Properties of the non-normalized cDNA samples. A. PCR amplification of the cDNA samples. B. Analysis of the PCR-amplified 3′-fragment cDNAs on the Shimadzu MultiNA microchip electrophoresis system. C. Analysis of the size-fractionated cDNAs on the Shimadzu MultiNA microchip electrophoresis system. D. Description of cDNA for Illumina sequencing. Illumina adapter sequences are underlined. M – 100 bp ladder; ISR – initiating storage root sample; FR – fibrous root sample. [file 1471-2164-14-460-S14.pdf]

A

Sample	ISR	FR
PCR cycles	15	19
5' Barcode	GAGT	CTTG
Concentration (ng/µl)	22	26
Volume (µl)	20	20

B

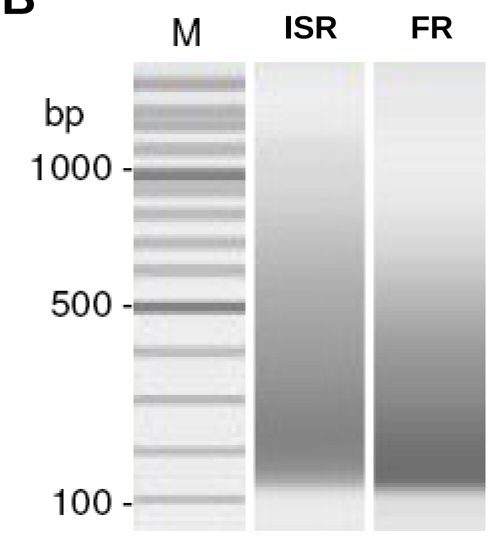

C

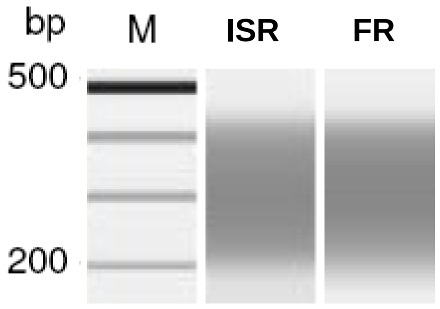

D

5'- end (53 bases): Tag sequence
5´- AATGATACGGCGACCACCGACAGGTTCAGAGTTCTACAGTCCGACGATC-NNNN-3'

3'-end (45 bases):
5'- CAAGCAGAAGACGGCATACGA-CCTGTCACTCACTGCGA(dT25)-3'
