# Supplementary material for: Transcriptional profiling of sweetpotato (Ipomoea batatas) roots indicates down-regulation of lignin biosynthesis and up-regulation of starch biosynthesis at an early stage of storage root formation
Source: BMC Genomics. 2013 Jul 9;14:460. doi: 10.1186/1471-2164-14-460 (PMC3716973; doi:10.1186/1471-2164-14-460)
Supplement: Additional file 15 — List of oligonucleotide primer sequences used for the quantitative RT-PCR analyses. [file 1471-2164-14-460-S15.pdf]

Gene	Contig	Primers
**18S**	-	F-CATGATAACTCGACGGAT<br>R-CTTGGATGTGGTAGCCGT
**class-I knotted1-like homeobox protein (IBKN2)**	S_PBL_c8137	F-GCCAGGCAGAAGTTGCTTAG<br>R-CAGTGCCGTTTTCTTTGGTT
**class-I knotted1-like homeobox protein (IBKN3)**	S_PBL_c31412	F-CGCCTAGGTCCATAATCCAA<br>R-TATTTCAAGGCGGTCTCAGG
**cyclin-dependent kinase A1**	S_PBL_c1272	F-ATGGTACAGGGCACCAGAAA<br>R-CTGGTTCACCATCTCAGCAA
**ADP-glucose pyrophosphorylase beta subunit (IbAGPb1A)**	S_PBL_lrc53818	F-GACAAGAACGTAAGGATTGGGA<br>R-CGAATGGTTGCTTTCTCCAT
**Granule-bound starch synthase 1**	S_PBL_c3042	F-AGGAAAACGTCGCTACTCCA<br>R-GTCCCCCATTGACTCTTTCA
**sporamin A precursor**	S_PBL_c2971	F-CACCAACAAGCTCTGCGTAA<br>R-GGCATCGACAACCTCAATCT
**coumaroyl-CoA synthase 1**	S_PBL_c158	F-TCCAGCTTCTAGCCTTGCTC<br>R-GCAGGAGAAGTTCCAGTTGC
**Caffeoyl-CoA O-methyltransferase**	S_PBL_c20480	F- AGGTTGGTGGTCTGATTGGA<br>R- GGGAAGTTGGCAAATCTCAA
**cinnamyl alcohol dehydrogenase**	S_PBL_lrc53688	F- GTCTTGGCGCAGACTCTTTC<br>R- TAATGGCACAACAGCGTGAT
